# Antifungal susceptibility, molecular epidemiology, and clinical risk factors of *Candida glabrata* in intensive care unit in a Chinese Tertiary Hospital

**DOI:** 10.3389/fcimb.2024.1455145

**Published:** 2024-10-07

**Authors:** Si-Jia Huang, Geng Lv, Yi-Hui Song, Jun-Tao Zhao, Jin-Yan Liu, Lu-Ling Wang, Ming-Jie Xiang

**Affiliations:** ^1^ Department of Laboratory Medicine, Ruijin Hospital, Shanghai Jiao Tong University School of Medicine, Shanghai, China; ^2^ Department of Laboratory Medicine, Ruijin Hospital Luwan Branch, Shanghai Jiao Tong University School of Medicine, Shanghai, China; ^3^ The Shanghai Institute of Hypertension, Ruijin Hospital, Shanghai Jiao Tong University School of Medicine, Shanghai, China

**Keywords:** *Candida glabrata*, intensive care unit (ICU), antifungal susceptibility, multilocus sequence typing (MLST), echinocandin resistance, risk factors

## Abstract

**Background:**

The increasing incidence and high mortality rate of *Candida glabrata* infection in ICU patients is an important issue. Therefore, it is imperative to investigate the antifungal susceptibility profiles and epidemiological characteristics in local regions.

**Methods:**

Herein, antifungal susceptibility testing was conducted to determine the minimum inhibitory concentrations (MICs) of eight antifungal drugs. Multilocus sequence typing (MLST) was used to study the strain genotype, geographical distribution, and susceptibility to antifungal agents among *C. glabrata* isolates. The mechanism of echinocandin resistance was explored by sequencing the *FKS1* and *FKS2* genes (encoding 1,3-β-D-glucan synthases) of echinocandin-resistant *C. glabrata* strains. Moreover, we further investigated the clinical manifestations and the various risk factors of patients infected with *C. glabrata* in the ICU.

**Results:**

We selected 234 C*. glabrata* isolates from 234 patients in the ICU randomly for the follow-up study. Cross-resistance was found among the ICU *C. glabrata* isolates. Analysis using MLST showed that the genetic diversity among the *C. glabrata* isolates was low. Furthermore, sequence type showed no correlation with the antifungal resistance profiles, but was associated with geographical distribution. We also revealed novel mutations in FKS1 (S629P) and FKS2 (W1497stop) that mediated high-level echinocandin resistance (MIC >8 µg/mL). More than 14 days’ stay in ICU (P=0.007), Acute Physiology and Chronic Health Evaluation II (APACHE-II) score (P=0.024), prior antifungal exposure (P=0.039) and lung disease (P=0.036) were significantly associated with antifungal resistant/non-wild-type *C. glabrata* infection.

**Conclusion:**

Our study shed light on the antifungal susceptibility, molecular epidemiology, and clinical risk factors of *C. glabrata* in the ICU of a Chinese Tertiary Hospital. Importantly, we revealed the molecular mechanism of echinocandin resistance. These results highlight the significance of continued surveillance in ICUs and provide data support for the treatment of *C. glabrata* in clinics.

## 1 Introduction

Species in the genus *Candida* comprise significant opportunistic pathogens, colonizing the oral cavity, vagina, or gastrointestinal tract of the majority of healthy individuals as part of their normal flora (Jensen et al., 2015; Barantsevich and Barantsevich, 2022). However, life-threatening systemic infections by *Candida* spp. are associated with specific factors that impair immune function or disrupt the microbiome, such as antibiotic therapy, cancer, or immunosuppression. *C. albicans* comprises the main infectious agent; however, there has been a progressive increase in the incidence of *Candida glabrata* infection over the years (Hassan et al., 2021), accompanied by a higher prevalence of drug-resistant strains, thus representing an alarming trend (Guinea, 2014; Díaz-García et al., 2021; McCarty et al., 2021). *C. glabrata* is the dominant candidemia-causing pathogen among *non-albicans* species in Australia and Malaysia (Chapman et al., 2017; Farooq et al., 2022).

Recently, echinocandins have been recommended as first-line treatments for *C. glabrata* infections because of this species’ decreased susceptibility to triazoles (Pappas et al., 2016; Czajka et al., 2023). However, recent years have seen the emergence of echinocandin resistance, with the highest rate being observed in *C. glabrata* (Alexander et al., 2013).The emergence of acquired resistance in *C. glabrata* towards echinocandins necessitates antifungal susceptibility testing as an essential tool to guide treatment decisions. Echinocandins inhibit the synthesis of the fungal cell wall by binding to the multiple subunits of the 1,3-β-D-glucan synthase complex, encoded by *FKS1*, *FKS2*, and *FKS3* in *C. glabrata*. Clinical studies have demonstrated that substitutions of amino acids in the hotspot-1 and hotspot-2 regions of the FSK1 and FSK2 subunits are responsible for conferring echinocandin resistance in *C. glabrata* strains (Perlin, 2015; Arastehfar et al., 2020). However, mutations outside the hotspot regions also have been reported (Hou et al., 2019).In patients lacking common risk factors associated with echinocandin resistance development, *FKS* mutation detection is currently reckoned to be the most accurate technique to predict treatment failure. Unfortunately, the molecular mechanism of echinocandin resistance remains to be determined.

Molecular typing of local strains is of great importance for epidemiological investigations and transmission prevention. Genotyping aims to trace the source of an infection, identify the transmission routes, and determine the extent of an epidemic (Chen et al., 2022). Numerous genotyping methods have been reported, such as microsatellite analysis, pulsed field gel electrophoresis (PFGE), restriction fragment length polymorphism (RFLP), and multilocus sequence typing (MLST) (Foulet et al., 2005; Lin et al., 2007; Canela et al., 2021). Among these techniques, MLST is widely used. This method differentiates strains by analyzing single nucleotide polymorphisms (SNPs) in fragments of housekeeping genes, being very reproducible and possessing high discriminatory power. The *C. glabrata* MLST system has been utilized since 2003, and involves comparisons of six housekeeping genes (*FKS*, *LEU2*, *NMT1*, *TRP1*, *UGP1*, and *URA3*) (Dodgson et al., 2003).

Patients in the intensive care unit (ICU) usually suffer from disrupted immunity and physiological dysfunction, likely due to their underlying diseases, invasive clinical interventions, hormone therapy, or length of hospital stay (Poissy et al., 2020). Consequently, they are susceptible to *C. glabrata* infection (Logan et al., 2020). *C. glabrata* infection in ICU patients is an important issue because of its increasing incidence and high mortality rate (Ostrosky-Zeichner and Al-Obaidi, 2017). The timely and rational utilization of antifungal agents is important for the prognosis of patients in the ICU. Therefore, it is imperative to assess the antifungal susceptibilities and epidemiological characteristics of *C. glabrata* infection in local regions, which can enhance early treatment strategies and subsequently improve clinical outcomes for patients in the ICU.

Herein, using related clinical data, the correlation between strain genotype, geographical distribution, and antifungal agent susceptibility among *C. glabrata* isolates in the ICU of a Tertiary Hospital in East China were evaluated. Meanwhile, we determined the sequences of *FKS1* and *FKS2* from *C. glabrata* strains that were resistant to echinocandins to investigate the molecular mechanism of echinocandin resistance. We further investigated the risk factors and clinical features of *C. glabrata*-infected ICU patients.

## 2 Materials and methods

### 2.1 Clinical isolates, growth conditions, and strain identification

A total of 589 clinical *C. glabrata* isolates were collected from January 2019 to December 2023 from the clinical laboratory in Ruijin Hospital, Shanghai Jiao Tong University School of Medicine, a tertiary teaching hospital comprising more than 3,000 beds. Different Isolates collected from the same patient were removed from the analysis. All the patients were from the ICU. Finally, 234 C*. glabrata* isolates from 234 patients were randomly selected for the follow-up study. All isolates were added with glycerol and stored at −80°C.

Matrix-assisted laser desorption/ionization time-of-flight mass spectrometry (MALDI-TOF MS) (Vitek MS, bioMérieux, Marcy l’Etoile, France) was used to identify all the clinical isolates, which were confirmed by sequencing of rDNA internal transcribed spacer (ITS) regions (Zhang et al., 2014). The ITS regions were amplified and sequenced using primers ITS1 and ITS4 (ITS1:5’-TCCGTAGGTGAACCTGCG-3’; ITS4:5’-TCCTCCGCTTATTGATATGC-3’). Species were identified (> 99% similarity) using BLAST searches (https://blast.ncbi.nlm.nih.gov/Blast.cgi).

The study was approved by the Ethics Committee of Ruijin Hospital, Shanghai Jiaotong University School of Medicine, with the approval number of MX-B4621R. The Ethics Committee waived the need to request informed consent from patients. Because this was a retrospective and observational study, and it did not have any research procedure affecting the patients’ health and safety adversely.

### 2.2 Testing for antifungal susceptibility

A colorimetric microdilution panel, DL-96Fungus (Dier Biotech, Guangzhou, China), was used for the *in vitro* susceptibility tests. Strains were grown for 24h at 37°C to determine the minimum inhibitory concentrations (MICs). A change in color from blue (negative, no growth) to red (positive, growth) indicated yeast growth. The quality control species comprised *Candida parapsilosis* ATCC 22019 and *Candida krusei* ATCC 6258. The Clinical and Laboratory Standards Institute (CLSI) M27M44S guidelines for azoles and echinocandins were used to interpret the MIC values (CLSI, 2022b). The resistance breakpoint to fluconazole was MIC ≥ 64 µg/mL, to caspofungin was MIC ≥ 0.5 µg/mL, and to micafungin was MIC ≥ 0.25 µg/mL (CLSI, 2022b). There are no CLSI-determined clinical breakpoints for amphotericin B, 5-flucytosine, posaconazole, itraconazole, and voriconazole; therefore, isolates were defined as wild-type (WT) or non-wild-type (non-WT) according to the epidemiological cut-off values (ECVs) (CLSI, 2022a). The non-WT ECV to voriconazole was MIC >0.25 µg/mL, to itraconazole was MIC >4 µg/mL, to posaconazole was MIC >1 µg/mL, to 5-flucytosine was MIC >0.5 µg/mL, and to amphotericin B was MIC >2 µg/mL (CLSI, 2022a).

### 2.3 Multilocus sequence typing

Genomic DNA was extracted from pure cultures using a TIANamp Yeast DNA Kit (Tiangen biotech, Beijing, China) following the manufacturer’s instructions. We studied six housekeeping gene loci (*FKS, LEU2, NMT1, TRP1, UGP1*, and *URA3*). The extracted genomic DNA was used as the substrate for PCR. The standardized MLST scheme-derived primers were employed to PCR amplify the gene fragments. PrimeSTAR^®^ HS DNA Polymerase (Takara, Shiga, Japan) was used to carry out the PCR reaction. Please refer to **Table 1** for a list of the primers used for amplification (Dodgson et al., 2003). The PCR reaction was carried out as follows: initial denaturation for 10 s at 98°C; 30 cycles of denaturation (10 s at 98°C), annealing (10 s at 50–60°C), and extension (1 min at 72°C); and a final extension at for 4 min 72 °C. Each PCR reaction (25µL) included 5 µL of 5×PrimeSTAR buffer (Mg^2+^ Plus), 2 µl of the dNTP mixture (2.5 mM each), 1 µL of the DNA template, 1 µL of the forward primer, 1 µL of the reverse primer, and 0.25 µL of PrimeSTAR HS DNA polymerase (2.5U/µL). Genewiz (Suzhou, China) sequenced the resultant amplicons in both directions using the same primers as were used for the PCR reaction. After manual analysis, the nucleotide sequences were carefully screened to ensure high-quality sequences. Subsequently, we used the sequences to query the *Candida glabrata* MLST database (https://pubmlst.org/organisms/candida-glabrata) to identify the corresponding allele at each locus. The allelic profiles of the isolates were used to define the sequence types (STs). For each new ST, the new allele type was sequenced twice in both directions for confirmation. We submitted the new alleles and STs to the MLST database, which were named by the database curator after scrutiny.

**Table 1:** Primers used for MLST of *C. glabrata*.

Locus	Primer	Sequence (5’—3’)	Annealing temperature(°C)
FKS	Forward	GTCAAATGCCACAA CAACAACCT	55.0
	Reverse	AGCACTTCAGCAG CGTCTTCAG	
LEU2	Forward	TTTCTTGTATCCTC CCATTGTTCA	54.0
	Reverse	ATAGGTAAAGGTG GGTTGTGTTGC	
NMT1	Forward	GCCGGTGTGGTG TTGCCTGCTC	59.0
	Reverse	CGTTACTGCGGTG CTCGGTGTCG	
TRP1	Forward	AATTGTTCCAGC GTTTTTGT	50.0
	Reverse	GACCAGTCCAGCT CTTTCAC	
UGP1	Forward	TTTCAACACCGAC AAGGACACAGA	57.0
	Reverse	TCGGACTTCACTAG CAGCAAATCA	
URA3	Forward	AGCGAATTGTTGAA GTTGGTTGA	53.0
	Reverse	AATTCGGTTGTAAG ATGATGTTGC	

### 2.4 Sequence analysis of *FKS1* and *FKS2*

We amplified the full-length DNA sequences of *FKS1* (5592 bp) and *FKS2* (5694 bp) employing the PCR primers provided in the previous study (Hou et al., 2019). The amplification program was performed as described previously. Agarose gel electrophoresis was used to semi-quantify the PCR products, which were then purified using a E.Z.N.A^®^ Gel Extraction Kit (Omega Laboratories Inc., Mogadore, OH, USA) according to the manufacturer’s instructions. The purified amplicons were sequenced by Genewiz in both directions and the results were analyzed using Snapgene 7.2 software (GSL Biotech LLC, Boston, MA, USA). The *FKS1* and *FKS2* sequences were compared with the reference wild-type (WT) *Candida glabrata* ATCC 90030 sequences (*FKS1*, GenBank accession number: HM366440.1; *FKS2*, GenBank accession number: HM366442.1).

### 2.5 Clinical data analysis

We analyzed the patients’ medical records retrospectively, which included demographic data, ICU length of stay, underlying diseases, healthcare factors, APACHE-II (Acute Physiology and Chronic Health Evaluation II) score, and outcome of therapy. The APACHE-II score was calculated by adding up the total acute physiology score, age points, and chronic health points. According to the antifungal resistance profiles of *C. glabrata* determined in section 2.2, we divided patients into two groups: the antifungal resistant/non-WT group and the antifungal non-resistant/WT group. Strains demonstrating resistance to any drug were classified as resistant/non-WT.

### 2.6 Statistical analysis

The antifungal resistance of all the eight drugs was assessed using SPSS software (version 22.0; IBM Corp., Armonk, NY, USA). Cluster analysis was employed to determine the genetic relationships among the isolates incorporating the minimum-spanning tree in the PHYLOViZ 2.0 software (Nascimento et al., 2017). The dendrogram was constructed using MEGA X (Tamura et al., 2021) based on Unweighted Pair Group Method with Arithmetic Mean (UPGMA). The clonal complexes (CCs) were distinguished using the goeBURST algorithm in PHILOVIZ 2.0 based on the MLST results. Variations in clinical risk factors were compared using the χ^2^ test. Statistical significance was considered at a P value of 0.05 or less.

## 3 Results

### 3.1 Characterization of *C. glabrata* isolates

We collected 234 C*. glabrata* isolates from patients in the ICU in Ruijin Hospital during 2019–2023. Each isolate was obtained from a different patient. 17.09% (40/234) were from 2019, 15.81% (37/234) were from 2020, 21.79% (51/234) were from 2021, 22.22% (52/234) were from 2022, and 23.08% (54/234) were from 2023. The patients comprised 124 men (52.99%) and 110 women (47.01%). Their average age was 67 ± 19.0 years and their median age was 69 years (range 20–98 years). Specimens were obtained mainly from urine (n = 74, 31.62%), shunt fluid (n = 37, 15.81%), and sputum (n = 35, 14.96%). The **Supplementary Table S1** provides the individual clinical characteristics of the cohort.

### 3.2 Patterns of antifungal susceptibility

**Table 2** shows the susceptibility of the 234 C*. glabrata* isolates toward the 8 tested antifungal drugs. All the clinical *C. glabrata* isolates were WT to amphotericin B. However,14.53% (34/234) of the isolates were resistant to fluconazole, while the remaining isolates were susceptible-dose dependent (SDD). Caspofungin resistance only appeared in five isolates. All the micafungin resistant isolates (n = 3) also exhibited resistance to caspofungin. Notably, one of them (RJ097) showed a strong caspofungin and micafungin resistant phenotype (MIC > 8 µg/mL). Applying the ECV, the non-WT rates of *C. glabrata* for 5-flucytosine, posaconazole, voriconazole and itraconazole were 1.28%,15.38%, 32.05%, and 7.69%, respectively. It was worth noting that 6.41% (15/234) of the isolates showed cross-resistance/non-WT to azoles (itraconazole, voriconazole, posaconazole, and fluconazole). One isolate (RJ162) showed resistance/non-WT to five drugs (caspofungin, voriconazole, posaconazole, fluconazole, and itraconazole) (**Supplementary Table S1**).

**Table 2:** Antifungal susceptibility results of all the 234 C*. glabrata* clinical strains in ICU.

Antifungal Agent	MIC(μg/mL)			Isolates(%)			Isolates(%)		
	Range	MIC_50_	MIC_90_	S	SDD	I	R	WT	non-WT
AmphotericinB	0.12-16	0.5	0.5	NA	NA	NA	NA	100	0
Flucyctosine	0.12-64	≤0.12	≤0.12	NA	NA	NA	NA	98.72	1.28
Micafungin	0.008-8	≤0.008	≤0.008	98.29	NA	0.43	1.28	NA	NA
Caspofungin	0.008-8	0.12	0.25	78.63	NA	19.23	2.14	NA	NA
Fluconazole	0.12-128	8	64	NA	85.47	NA	14.53	NA	NA
Voriconazole	0.015-16	0.25	2	NA	NA	NA	NA	67.95	32.05
Posaconazole	0.015-16	0.5	2	NA	NA	NA	NA	84.62	15.38
Itaconazole	0.03-16	0.5	2	NA	NA	NA	NA	92.31	7.69

MIC, minimum inhibitory concentration; S, susceptible; SDD, susceptible-dose dependent; I, intermediate; R, resistant; WT, wild-type; non-WT, non-wild-type; NA, not applicable.

**Figure 1:**
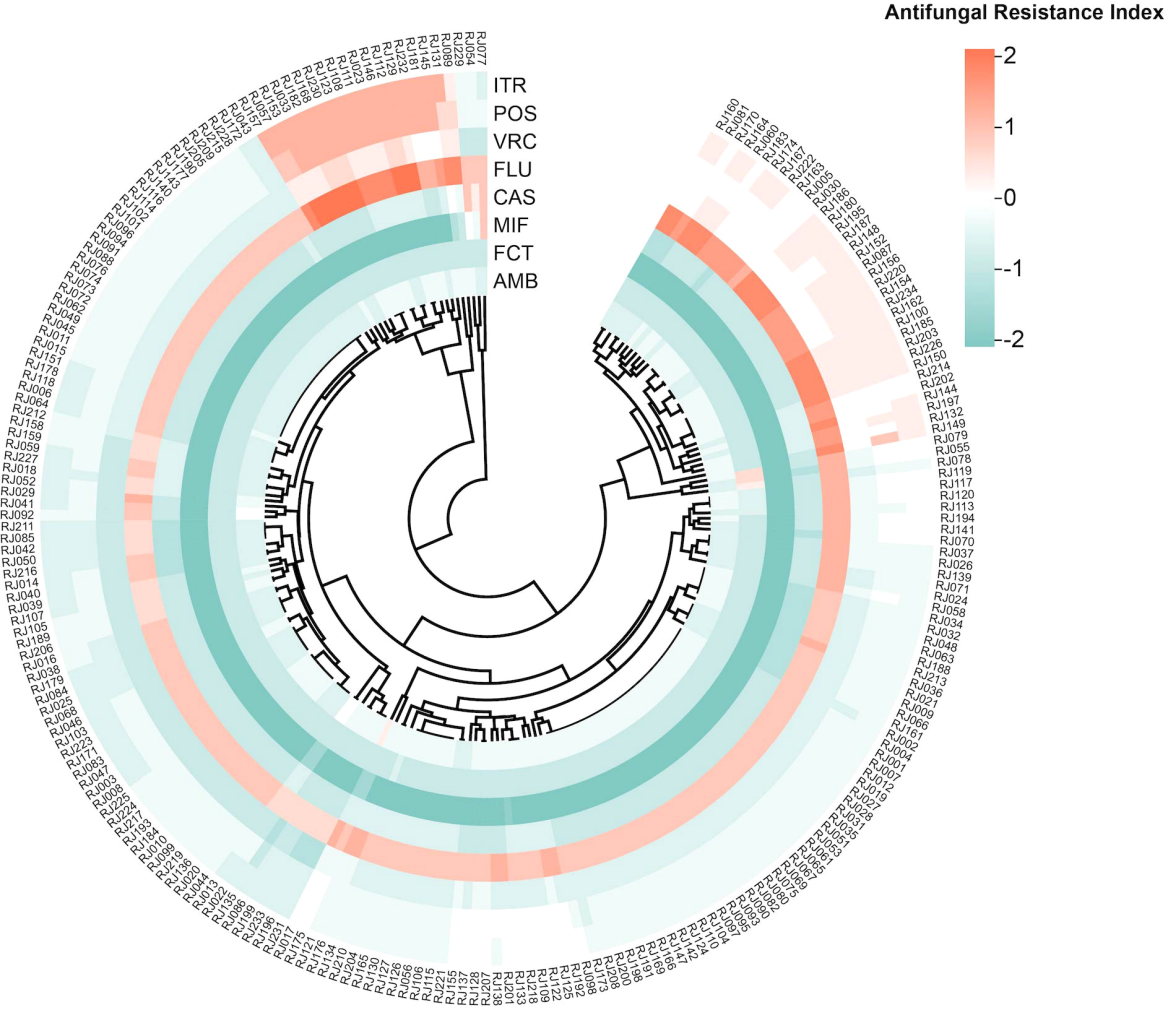
Heat map of resistance-level based on antifungal resistance patterns. The heat map shows the antifungal resistance index for different antifungal drugs of each strain. Each row represents an antifungal drug and each column refers to a clinical isolate. The outermost circle displays the strain number. The drug resistance index is calculated as the logarithm of the MIC value obtained from antifungal susceptibility results. Abbreviations: AMB, Amphotericin B; FCT, 5-Flucytosine; MIF, Micafungin; CAS, Caspofungin; FLU, Fluconazole; VRC, Voriconazole; POS, Posaconazole; ITR, Itraconazole.

The drug resistance index was determined using the logarithm of the MIC value, as shown in the heat map (**Figure 1**). Large to small log (MIC) values are indicated by red to green colors, with a larger contrast between the colors indicating a larger disparity in the MIC of antifungal agents. As can be seen, the azoles area is redder, while the echinocandins area is greener. The heat map of the resistance index demonstrated more susceptibility of *C. glabrata* isolates from ICU patients to echinocandins and a trend of stronger resistance to azoles.

### 3.3 MLST analysis

**Figure 2:**
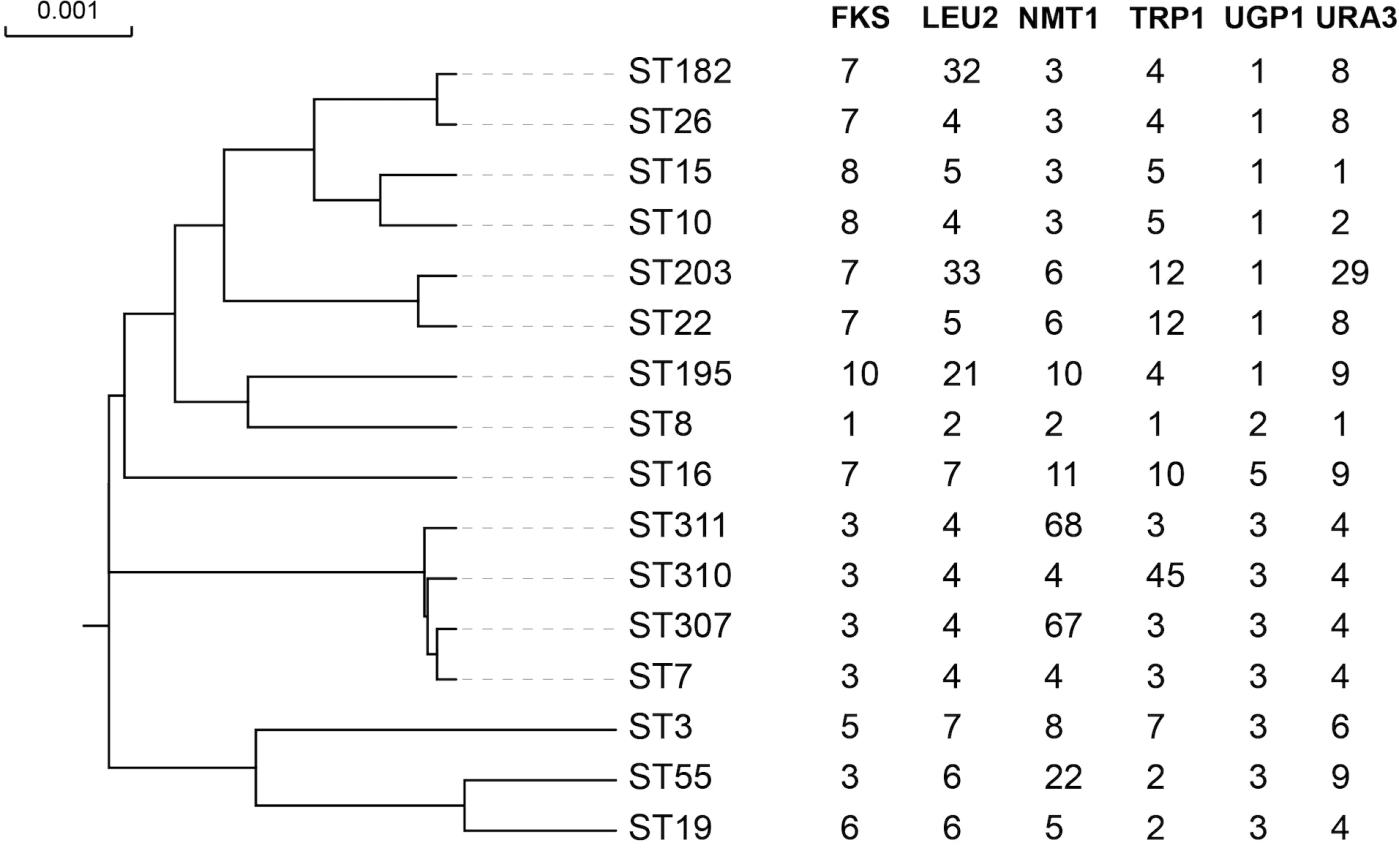
The phylogenetic tree of all the ST types based on UPGMA. The full length of six alleles’ sequence was included into the alignment. The highly similar sequence was observed between ST55 and ST19, ST307 and ST7, ST195 and ST8, ST203 and ST22, ST182 and ST26, and ST15 and ST10. The other STs showed a high genetic diversity.

Overall, the *C. glabrata* isolates showed low genetic diversity according to the MLST analysis in **Table 3**. *NMT1* exhibited the highest typing efficiency, with 11 distinct alleles, whereas *UGP1* displayed the lowest typing efficiency, with only 4 distinct alleles. The six polymorphic alleles (*FKS, LEU2, NMT1, TRP1, UGP1*, and *URA3*) combined to form 16 different STs among the 234 isolates. The predominant ST type was ST7 (164/234, 70.09%), followed by ST10 (22/234, 9.40%), ST15 (14/234, 5.98%), and ST3 (9/234,3.85%). Three new STs (ST307, ST310, and ST311) were found. Three novel alleles were identified: Two in *NMT1* (allele 67 and allele 68) and one in *TRP1* (allele 45). We have submitted the new ST and alleles to the *C. glabrata* MLST database (https://pubmlst.org/organisms/candida-glabrata). To evaluate the genetic distance among the STs, a dendrogram (**Figure 2**) based on the UPGMA analysis was created, which showed that highly similar sequences were observed between ST55 and ST19, ST307 and ST7, ST195 and ST8, ST203 and ST22, ST182 and ST26, and ST15 and ST10. High genetic diversity was observed among the remaining STs.

**Table 3:** Summary of genotypes for 234 C*. glabrata* isolates using MLST.

ST	Isolates (Number)	*FKS*	*LEU2*	*NMT1*	*TRP1*	*UGP1*	*URA3*
7	164	3	4	4	3	3	4
10	22	8	4	3	5	1	2
15	14	8	5	3	5	1	1
3	9	5	7	8	7	3	6
19	4	6	6	5	2	3	4
55	3	3	6	22	2	3	9
182	3	7	32	3	4	1	8
**307**	3	3	4	**67**	3	3	4
22	2	7	5	6	12	1	8
26	2	7	4	3	4	1	8
195	2	10	21	10	4	1	9
**311**	2	3	4	**68**	3	3	4
8	1	1	2	2	1	2	1
16	1	7	7	11	10	5	9
203	1	7	33	6	12	1	29
**310**	1	3	4	4	**45**	3	4

The alleles and sequence types (STs) highlighted in bold were novel ones found in this study.

### 3.4 Relationships between MLST genotyping, antifungal susceptibility, and geographical distribution

To provide a more intuitive display of the antifungal resistance pattern, we selected five drugs (amphotericin B, caspofungin, fluconazole, voriconazole, and itraconazole) that are commonly used in the clinic for further research and classification. The classification results are listed in **Table 4**. Above all, 156 isolates were non-resistant/WT to all five drugs. The remaining 78 isolates were divided into five classes (C); Class 2 and Class 5 were further categorized into Group1 and Group 2. Class 1 (C1) contained 38 isolates with a WT, non-resistant, SDD, non-WT, and WT phenotype to the five antifungals (amphotericin B, caspofungin, fluconazole, voriconazole, and itraconazole), respectively; Class 2 Group 1 (C2 G1) contained 18 isolates with a resistant and a non-WT phenotype to fluconazole and voriconazole; Class 2 Group 2 (C2 G2) contained 3 isolates with an SDD, non-WT and non-WT phenotype to fluconazole, voriconazole, and itraconazole, respectively; Class 3 (C3) comprised 14 isolates that were resistant to fluconazole, and showed a non-WT phenotype to voriconazole and itraconazole; Class 4 (C4) contained 3 isolates that were resistant to caspofungin but showed an SDD phenotype to fluconazole; Class 5 Group 1 (C5 G1) contained 1 isolate that was resistant to caspofungin and fluconazole, and showed a non-WT phenotype to voriconazole and itraconazole; and Class 5 Group 2 (C5 G2) contained 1 isolate that was resistant to caspofungin and fluconazole, and showed a non-WT phenotype to voriconazole.

**Table 4:** Antifungal susceptibility patterns and multilocus sequence typing (MLST) genotypes of 234 C*. glabrata* isolates.

Antifungal susceptibility pattern (AMB,CAS,FLU,VRC,ITR)	MLST sequence type (ST)	Isolates (Number)
Non-resistant (WT,non-R,SDD,WT,WT)	ST7	97
	ST10	16
	ST15	11
	ST3	8
	ST19	4
	ST55,ST182,ST307	3
	ST26,ST195,ST310	2
	ST8,ST16,ST22,ST203,ST311	1
C1 (WT,non-R,SDD,NWT,WT)	ST7	31
	ST10	5
	ST15,ST22	1
C2 G1 (WT,non-R,R,NWT,WT)	ST7	17
	ST3	1
C2 G2 (WT,non-R,SDD,NWT,NWT)	ST7	3
C3 (WT,non-R,R,NWT,NWT)	ST7	12
	ST10,ST15	1
C4 (WT,R,SDD,WT,WT)	ST7	2
	ST15	1
C5 G1 (WT, R,R,NWT,NWT)	ST7	1
C5 G2 (WT, R,R,NWT,WT)	ST7	1

**Figure 3:**
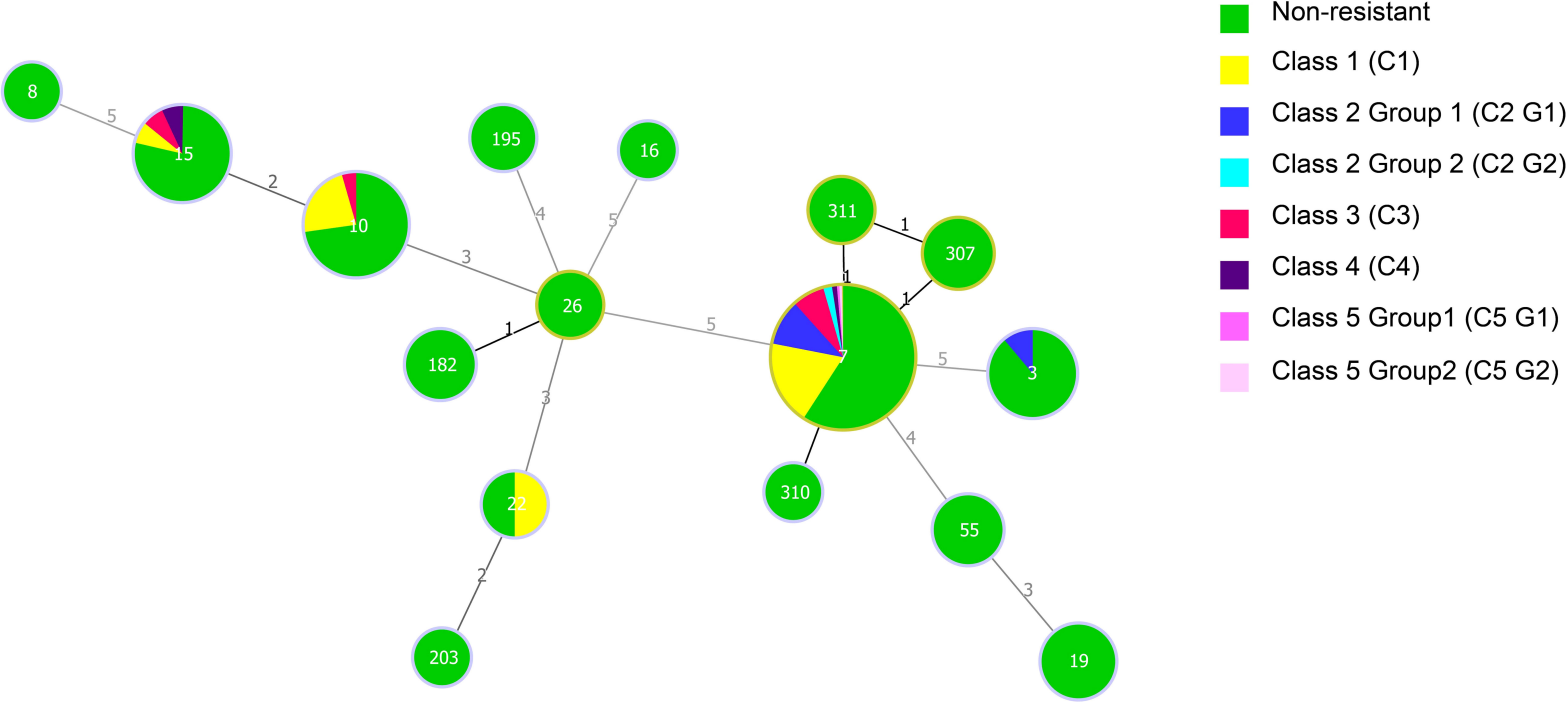
Minimum spanning tree of 234 C*. glabrata* isolates conducted by MLST analysis via the PHYLOViZ 2.0 software. The relationship between the sequence types (STs) and the patterns of antifungal resistance (amphotericin B, caspofungin, fluconazole, voriconazole, and itraconazole) was assessed. Each circle corresponds to a ST genotype while the circle size indicates the number of isolates. The number on the line shows the count of allelic differences between two STs.

Analysis using Minimum Spanning Tree (MST) determined the association between the MLST genotypes and the patterns of antifungal resistance (**Figure 3**). The most prevalent ST7 genotype contained all kinds of antifungal resistance patterns (C1–C5). Of the five caspofungin-resistant C. glabrata isolates which belong to C4 and C5, four isolates had the ST7 genotype and the other had ST15. Moreover, 1 ST3 isolate and 18 ST7 isolates showed resistance/non-WT to fluconazole and voriconazole. In addition, another 13 ST7 isolates, 1 ST10 isolate and 1 ST15 isolate also exhibited non-WT to itraconazole. In terms of isolates showing cross-resistance to azoles, ST7 was the dominant genotype.

Among the 78 isolates with a resistant/non-WT phenotype, 67 (85.90%) correlated with the predominant genotype, ST7, while the rest belonged to ST10 (6 isolates), ST15 (3 isolates), ST22 (1 isolate), and ST3 (1 isolate). 31 C1 isolates, 20 C2 isolates, 12 C3 isolates, 2 C4 isolates, and 2 C5 isolates all had the ST7 genotype. The non-resistant pattern contained all the 16 different STs, however, the resistant/non-WT phenotypes were only made up of 5 STs (ST3, ST7, ST10,ST15,ST22). Obviously, compared with that of the resistant/non-WT strains, the non-resistant strains had a greater genetic diversity.

**Figure 4:**
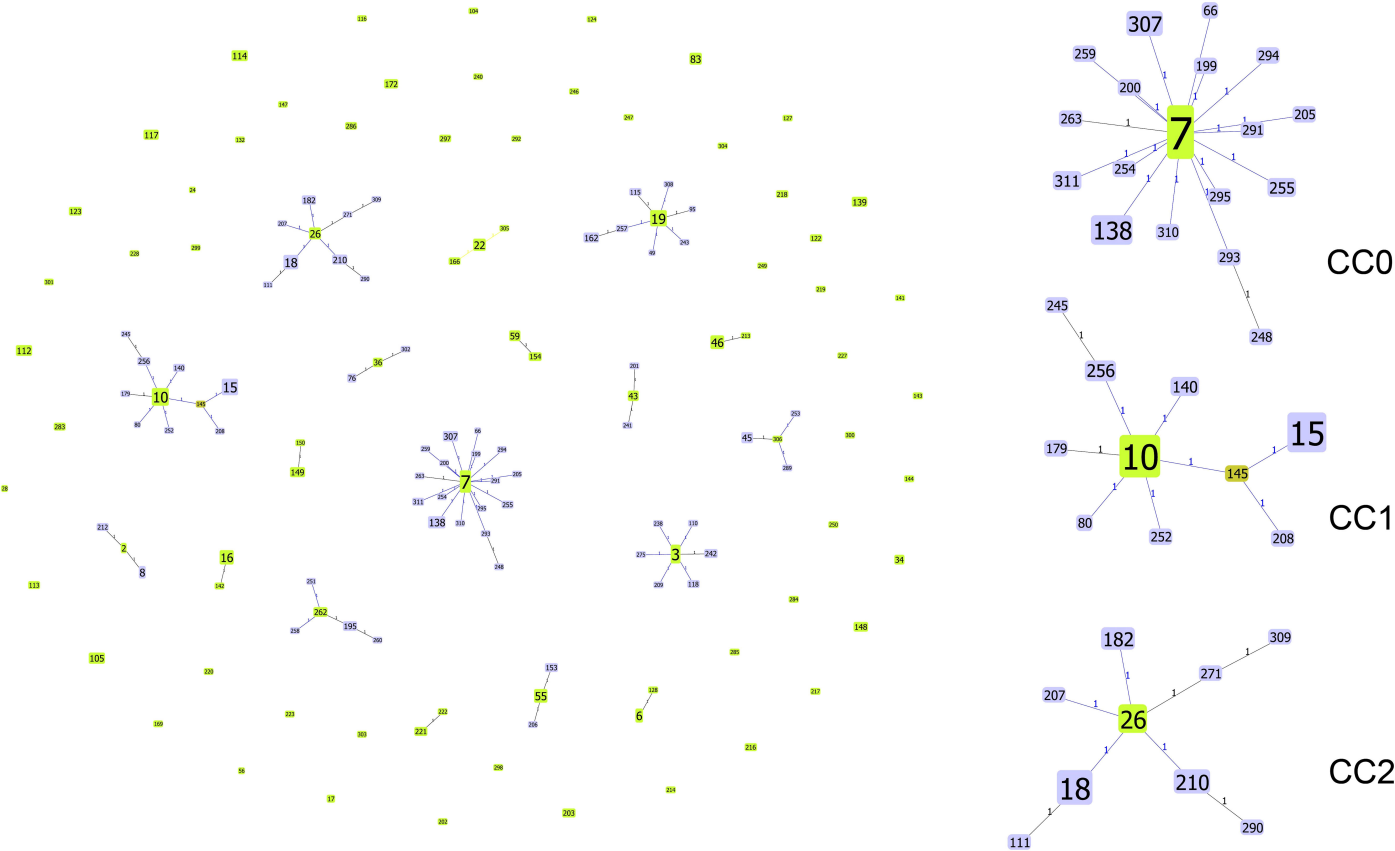
Genetic population structure of 1001 C*. glabrata* isolates. Each circle corresponds to a ST genotype while the circle size indicates the number of isolates. The number on the line shows the count of allelic differences between two STs. The color of the ST node refers to the genetic level (light green, group founder; dark green, sub-group founder; light blue, common node).The goeBURST analysis grouped 142 STs into 18 CCs and 54 singletons.

**Figure 5:**
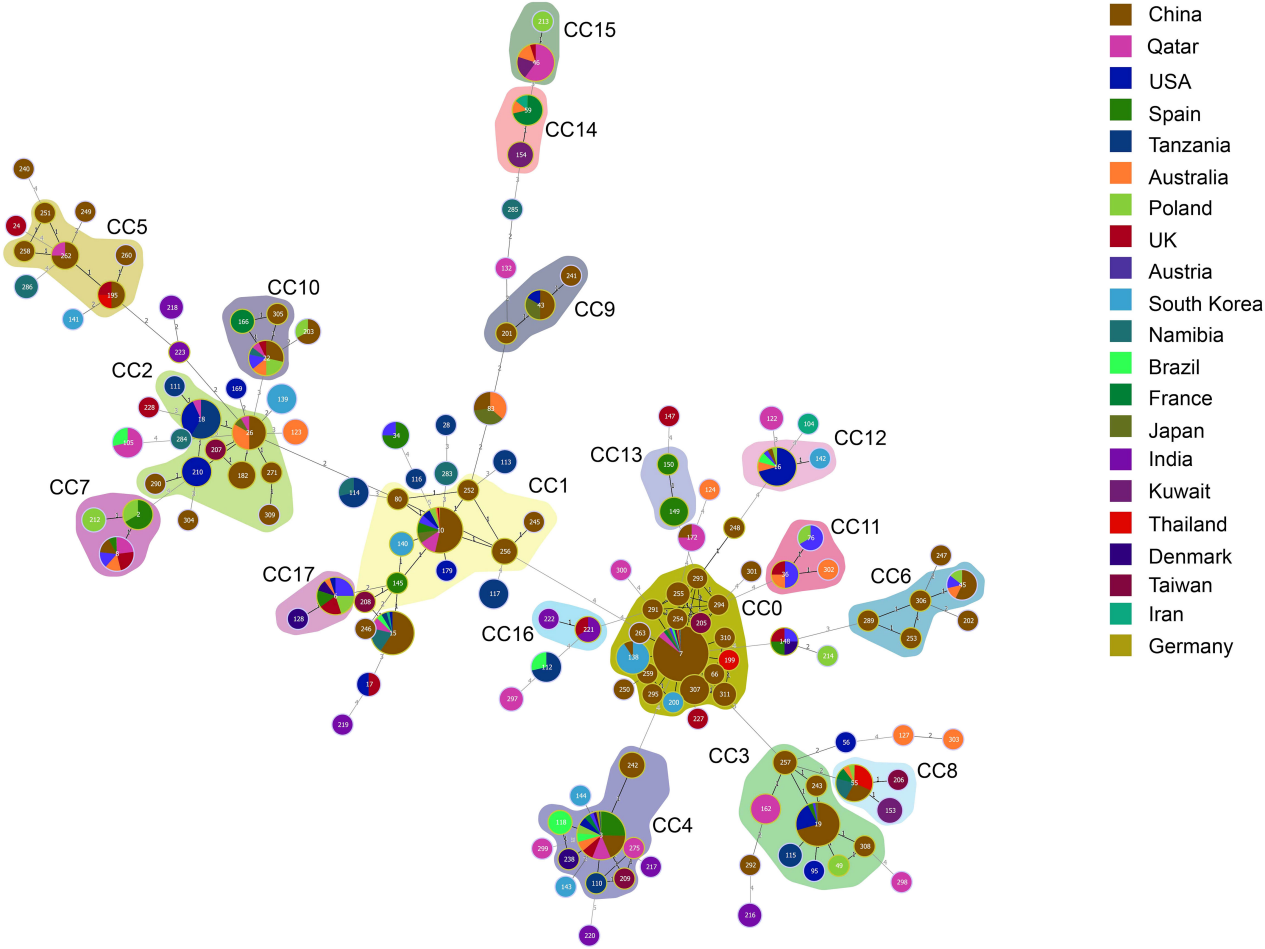
Minimum spanning tree of 1001 C*. glabrata* isolates. Each circle corresponds to a ST genotype while the circle size indicates the number of isolates. The color of the circle refers to the country to which it belongs. The shaded areas represent the groups of specific clone complexes (CC).

Next, the genetic relationships between the 234 local ICU clinical isolates and 767 strains deposited in the MLST database from 2014–2023 were evaluated. From these 1001 strains, goeBURST analysis grouped 142 STs into 18 CCs (CC0∼CC17) and 54 singletons (**Figure 4**). ST7 (31.07%,311/1001), ST3 (10.29%,103/1001), and ST10 (6.69%,67/1001) were the three most frequent ST types. CC0, CC1, and CC2 ranked the top three in terms of the number of STs, with each containing 18, 10 and 9 STs, respectively. Most strains (480/1001, 47.95%) were isolated in China. According to the minimum spanning tree (**Figure 5**), the region showed aggregation among different CC clone groups. China dominates in certain CCs such as CC0, CC3, CC5 and CC9. Notably, the majority of the Chinese isolates clustered with those from other Asian countries. On the other hand, 6 CCs (CC11, CC13, CC14, CC15, CC16, and CC17) among the 18CCs (CC0∼CC17) did not contain strains from China. In CC13, all six strains were derived from Spain (5 isolates were ST149 and 1 isolate was ST150).

As can been seen, from **Supplementary**-**Table S2**, the dominant ST genotype varied from country to country. ST7 accounted for 55.42% (266/480) in China, which is consistent with the results for *C. glabrata* isolates from ICU in Shanghai in this study, while ST3 was the predominant genotype in Spain, reaching proportions of 50.00% (26/52). However, ST16 ranked first in USA (26.98%, 17/63). ST18 was the top genotype in Tanzania (36.2%,17/47), and ST138 was more prevalent in South Korea (43.5%,10/23). Furthermore, it was found that there was only one strain for each of the 83 ST types. The result revealed a strong association between MLST typing and geographical distribution.

### 3.5 Detection of *FKS1* and *FKS2* mutations

To further investigate the molecular mechanisms of *C. glabrata* echinocandin resistance, the sequences of *FSK1* and *FSK2* from five echinocandin-resistant *C. glabrata* were obtained. The isolate sequences were compared with the full length *FKS1* (GenBank accession number: HM366440.1) and *FKS2* (GenBank accession number: HM366442.1) sequences from *C. glabrata*. Besides some synonymous mutations, multiple missense mutations were identified. The clinical details, the results of antifungal susceptibility testing for the four antifungal drugs commonly used in clinic and mutations in FKS1 and FKS2 are shown in **Table 5**. Remarkably, one of the five echinocandin-resistant *C. glabrata* isolates (RJ097) exhibited high resistance to caspofungin (MIC > 8 µg/mL). Both an S629P mutation in FKS1 and a premature stop codon (W1497stop) in FKS2 were discovered in this isolate, which have not been previously reported yet. For the other four isolates, the mutations were found in either FKS1 or FKS2 (three in FKS2 and one in FKS1). Two of them possessed the same single missense mutation (T1987C) in *FKS2*, leading to the amino acid substitution S663P. Another single missense mutation (C1999A) in *FKS2* resulted in the amino acid substitution P667T. In addition, one isolate harbored a nonsynonymous mutation in *FKS1* (T1885C), bringing about the amino acid substitution S629P.

**Table 5:** Characteristics of patients with echinocandin-resistant *C. glabrata*.

Patient			Confirmed disease	CAS treatment duration	Clinical source	Isolate no.	Sequence type (ST)	MIC(μg/mL) for				Mutation in FKS1&FKS2	
No.	Gender	Age						CAS	FLU	VRC	ITR	Nucleotide Change	Amino acid Change
1	F	85	Acute pancreatitis	16 days	Urine	RJ070	7	**2**	8	0.12	0.5	FKS1: Wild type FKS2: T1987C	FKS1: Wild type FKS2:S663P
2	F	85	Acute obstructive suppurative cholangitis	14 days	Urine	RJ097	7	**>8**	8	0.12	0.25	**FKS1:T1885C** **FKS2:G4491A**	**FKS1:S629P** **FKS2:W1497stop**
3	F	79	Sepsis	10 days	Urine, catheter	RJ111	7	**0.5**	**64**	**2**	2	FKS1: Wild type FKS2: T1987C	FKS1: Wild type FKS2: S663P
4	F	82	Severe pneumonia	8 days	Urine	RJ162	7	**1**	**64**	**2**	**>16**	FKS1: Wild type FKS2: C1999A	FKS1: Wild type FKS2:P667T
5	M	68	Acute necrotizing pancreatitis	17 days	Catheter	RJ270	15	**8**	8	0.12	0.5	FKS1:T1885C FKS2:Wild type	FKS1:S629P FKS2:Wild type

F, female; M, male; MIC, minimum inhibitory concentration; CAS, caspofungin; FLU, fluconazole; VRC, voriconazole; ITR, itraconazole.

MIC values indicative of resistance/non-wild-type to antifungal drugs are shown in bold.

The nucleotide and amino acid changes highlighted in bold have not been previously reported yet.

Two of the five caspofungin resistant *C. glabrata* isolates (RJ111&RJ162) exhibited a multidrug-resistant phenotype according to their cross resistance/non-WT to azoles. Five patients had available clinical details and a history of previous exposure to echinocandins. Four of the patients were elderly females (≥79 years). All the five patients had been treated for at least 8 days with caspofungin as treatment or prophylaxis. The *C. glabrata* strains were isolated from three patients’ urine samples and the other two were from urine, catheter and catheter (**Table 5**).

### 3.6 Clinical features of the ICU patients infected with *C. glabrata*

We analyzed the demographics, ICU length of stay, APACHE-II score, underlying diseases, healthcare factors, and clinical outcomes of 234 patients. As summarized in **Table 6**, staying in the ICU for more than 14 days (P=0.007), the APACHE-II score (P=0.024), prior antifungal exposure (P = 0.039) and lung disease (P=0.036) were significantly associated with antifungal resistant/non-WT *C. glabrata* infection. Besides, the other features of patients infected with antifungal resistant/non-WT and non-resistant/WT *C. glabrata* showed no statistically significant differences.

**Table 6:** Risk factors of *C. glabrata* in ICU between Antifungal non-resistant/WT group and Antifungal resistant/non-WT group.

Clinical characteristics	Antifungal non-resistant/WT group (n=156)	Antifungal resistant/ non-WT group (n=78)	P-value
Demographics			
Age			
Y, median n (%)	64.5	70.7	
0-14	0 (0)	0 (0)	
15-49	29 (18.6)	17 (21.8)	0.561
50-65	44 (28.2)	8 (10.3)	0.002
>65	83 (53.2)	53 (67.9)	0.031
Sex n (%)			
M	93 (59.6)	31 (39.7)	
F	63 (40.4)	47 (60.3)	
ICU length of stay n (%)			
<7 days	51 (32.7)	16 (20.5)	0.052
7-14 days	57 (36.5)	24 (30.8)	0.382
>14 days	48 (30.8)	38 (48.7)	0.007
APACHE-II			
APACHE-II score	16.07 ± 4.01	17.3 ± 3.66	0.024
Underlying diseases n (%)			
Cerebral disease	13 (8.3)	5 (6.4)	0.603
Diabetes	25 (16.0)	12 (15.4)	0.899
Tumor	23 (14.7)	9 (11.5)	0.501
Heart disease	15 (9.6)	10 (12.8)	0.454
Lung disease	90 (57.7)	56 (71.8)	0.036
Hepatobiliary disease	61 (39.1)	30 (38.6)	0.925
Kidney disease	33 (21.2)	12 (15.4)	0.291
Hypertension	29 (18.6)	15 (19.2)	0.906
Reproductive system disease	8 (5.1)	2 (2.5)	0.568
Healthcare factors n (%)			
Post-transplantation	9 (5.8)	5 (6.4)	0.922
Surgery	56 (35.9)	29 (37.2)	0.848
Mechanical ventilator	92 (59.0)	41 (52.6)	0.351
Indwelling urinary catheter	45 (28.8)	26 (33.3)	0.482
Central venous catheter	35 (22.4)	15 (19.2)	0.573
Prior antifungal exposure	123 (78.8)	70 (90.0)	0.039
Prior antimicrobial exposure	151 (96.8)	76 (97.4)	0.892
Clinical outcomes n (%)			
Improvement	79 (50.6)	33 (42.3)	0.229
Deterioration	61 (39.1)	40 (51.3)	0.076
Death	16 (10.3)	5 (6.4)	0.332

The variance between two groups was calculated with the χ2 test. A P value of 0.05 was considered significant.

## 4 Discussion

In this study, we explored the association between the profiles of antifungal susceptibility and the molecular epidemiology of *C. glabrata* in an ICU in Shanghai, China. We also studied the relationship between geographical distribution and *C. glabrata* genotypes. The molecular mechanisms of *C. glabrata* resistance to echinocandins were studied using sequencing analysis of *FKS1* and *FKS2*. Moreover, the risk factors of *C. glabrata* in the ICU were investigated between the antifungal resistant/non-WT group and the non-resistant/WT group.

In critically ill patients in the ICU, *C. glabrata* can manifest as colonization, isolated candidemia, invasive candidiasis (IC), or a mixed form of these three conditions (Hernández-Pabón et al., 2024). The incidence of *C. glabrata* infections among ICU-hospitalized patients has increased over the past decade and is associated with significant morbidity and mortality (Ostrosky-Zeichner and Al-Obaidi, 2017; Poissy et al., 2020; Wang et al., 2021). Nevertheless, relevant data remain scarce on the surveillance of *C. glabrata* isolation in ICUs in eastern China, especially their antifungal resistance profiles and genotypes.

There are three frequently used molecular typing tests for *C. glabrata*: PFGE, microsatellite length polymorphism (MLP), and MLST (Canela et al., 2021). Among them, MLST is commonly employed for the systematic phylogenetic and global epidemiological investigations of strains (Lin et al., 2007; Chen et al., 2022). Herein, we applied MLST to investigate the genetic relationships and epidemiology of *C. glabrata* infections in an ICU ward. A previous study identified 11 new STs among 91 C*. glabrata* isolates from 9 hospitals in Kuwait, which showed high genetic diversity (Asadzadeh et al., 2023). However, the present study identified only 16 STs among 234 isolates, with a ratio of STs to isolates of 14.6, which is similar to a multi-center study in China during 2009 to 2014 with a ratio of 11.7 (35 STs in 411 isolates) (Hou et al., 2017). In this study, *C. glabrata* displayed a low genetic diversity, with only three new alleles and three new STs, which might be attributed to the limited sample sources (from one specific ward in one hospital). Herein, ST7 was the dominant genotype detected in all the isolates, as well as in nearly all major specimen types, which is consistent with previous studies in other Asian regions, such as Taiwan (Lin et al., 2007), Japan (Khalifa et al., 2020) and Republic of Korea (Byun et al., 2018).

However, we cannot generalize the genetic variation among isolates in a particular region to other areas. By contrast, isolates from the USA (Lott et al., 2012) and Australia (Biswas et al., 2018) commonly comprise the ST3 genotype (38 of 201, 18.1%; and 8 of 51, 15.7%; respectively). To observe a geographical bias in the prevalence of circulating STs more clearly, we assessed the patterns of genotypes and geographical regions both in this study and the global data set (1001 isolates were finally included in the analysis). As expected, the prevailing ST types vary by regions. The ST7 was the predominant genotype in China, while other STs (ST16, ST18, and ST138) dominate in the particular regions. Notably, isolates from China accounted for a significant proportion of the top three ST types (ST7, ST3, and ST10). This might be attributed to the fact that the *C. glabrata* MLST database contains data for a substantial number of Chinese isolates. Importantly, these results complement the current prevalence characteristics of the different ST types of *C. glabrata* from Shanghai to the Global.

*C. glabrata* can be treated using antifungal drug classes including polyenes, echinocandins, and azoles (Perlin et al., 2017). Local epidemiology might be a useful reference to select the appropriate antifungal treatment (Ostrosky-Zeichner and Al-Obaidi, 2017). A comprehensive understanding of antifungal susceptibility patterns in the local region will help to improve patient management, especially in the treatment of critically infected patients (Perlin et al., 2017). The emergence of increased fluconazole resistance has led to echinocandins being recommended as first-line *C. glabrata* therapy (Pappas et al., 2016; Zeng et al., 2020). Herein, the susceptibility of *C. glabrata* to eight antifungal drugs was assessed. In agreement with the results of a large Chinese study from 2009 to 2014 (Hou et al., 2017), we observed 100% susceptibility to amphotericin B. Caspofungin resistance was displayed by only five strains, which corresponded to the previously reported scarcity of caspofungin resistance (Hou et al., 2017; Chen et al., 2022; Zhang et al., 2024). However, in the United States, echinocandin-resistant cases have been reported to reach as high as 10% (Pfaller et al., 2012; Pham et al., 2014). In line with our findings, several previous studies also described that *C. glabrata* isolates showed a rather high cross-resistance rates to azoles (Song et al., 2020; Chen et al., 2022; Zhang et al., 2024). Despite the relatively low overall resistance rates to amphotericin B, caspofungin, and azoles, emerging cross-resistance and multidrug resistance have been observed among *C. glabrata* strains, reinforcing the necessity for ongoing surveillance to determine trends in antifungal susceptibility (Song et al., 2020).

The results for the analysis of the antifungal susceptibility patterns and ST genotypes indicated that the most frequent ST types (ST7, ST10, ST15, ST3) possessed the most patterns, whether in non- resistant/WT or resistant/non-WT groups. The remaining ST types, such as ST55, ST182, and ST307, were exclusively present in the susceptibility group; however, their quantity (n < 5) is insufficient to yield significant results. By contrast, among the resistant/non-WT phenotype *C. glabrata* isolates, 67 out of 78 (83.3%) were the dominant ST7 genotype. The same result was observed in all the resistance patterns (C1–C5). Therefore, our findings agree with those of published studies (Klotz et al., 2016; Amanloo et al., 2018). Among *C. glabrata* isolates, the predominant genotype does not correlate with the antifungal resistance profile. However, in contrast to our results, Kiasat et al. (2019) (Kiasat et al., 2019) reported the predominant genotype (GT27) and the structures of *C. glabrata* populations were associated positively with antifungal drug resistance. Similar findings were reported by Dhieb et al. (2015) (Dhieb et al., 2015). This difference could be due to the clinical isolate sources or the contrasting genotyping assays used (MLST *vs*. microsatellite analysis).

The emerging echinocandin resistance in *C. glabrata* clinical isolates is a growing concern (Alexander et al., 2013). *In vitro* and in patients, echinocandin exposure can easily lead to the development of resistance (Bordallo-Cardona et al., 2017; Shields et al., 2019). Currently, *FKS* mutations are considered good predictors of a poor treatment response and echinocandin resistance in *C. glabrata* (Pham et al., 2014). Resistance is often mediated by point mutations in *FKS1* and *FKS2* hot-spot regions (Pfaller et al., 2012). In this study, five caspofungin-resistant *C. glabrata* were detected. Herein, S663P and P667T mutations were found in FKS2, while an S629P mutation was found in FKS1. Our data align with other reports (Zimbeck et al., 2010; Alexander et al., 2013), indicating that mutations in the FKS2 are more prevalent than those in the FKS1. The S663P mutation was commonly observed in echinocandin-resistant *C. glabrata* isolates from diverse geographic locations (Alexander et al., 2013; Castanheira et al., 2014). Importantly, both an S629P mutation in the FKS1 HS1 region and a premature stop codon (W1497stop) in FKS2 were discovered in the strain that exhibited high level caspofungin-resistance (MIC > 8 µg/mL), which has not been reported yet. Similarly, a previous research has proven that mutations in both FKS1 (W508stop) and FKS2 (E655K) were likely to mediate higher MICs to echinocandins than single mutations (Hou et al., 2019). However, the mechanism needs to be further verified. In addition, the development of caspofungin resistance was related closely to *in vivo* exposure. According to the clinical data, all five patients in this study were exposed to echinocandins for more than 8 days before isolation of the resistant strains. This underscores the need for careful consideration of the potential development of resistance when applying a long time echinocandin treatment or prophylaxis in ICU.

This study had some limitations. First, it was a retrospective, single-center study, although a large sample size of 234 C*. glabrata* isolates was studied. Second, only the molecular mechanisms of *C. glabrata* resistance to echinocandins were explored, while the mechanisms of resistance to azoles were not included because of financial constraints. What’s more, due to the limited experimental condition, we did not supplement mutational studies for the validation of molecular mechanisms of echinocandin resistance.

In summary, this study shed light on the antifungal susceptibility, molecular epidemiology, and clinical risk factors of *C. glabrata* in the ICU of a Chinese Tertiary Hospital. Cross-resistance was identified among in the *C. glabrata* isolates from the ICU. Meanwhile, ST type was proven to have no correlation with antifungal resistance profiles, but was associated with geographical distribution. More importantly, we revealed novel mutations in *FKS1* and *FKS2* that mediate high-level echinocandin resistance. Overall, our results highlight the importance of continued surveillance in ICUs and provide data support for the treatment of *C. glabrata* in clinics.

## Data Availability

The datasets presented in this study can be found in online NCBI database, accession numbers from PP908965 to PP908974.
