# Supplementary material for: Antifungal susceptibility, molecular epidemiology, and clinical risk factors of Candida glabrata in intensive care unit in a Chinese Tertiary Hospital
Source: Front Cell Infect Microbiol. 2024 Oct 7;14:1455145. doi: 10.3389/fcimb.2024.1455145 (PMC11491434; doi:10.3389/fcimb.2024.1455145)
Supplement: Supplementary file 1 [file Table1.docx]

Supplementary Table S1. Isolation time, clinical information (sex, age), sample, MLST analyses and antifungal susceptibility (MICs) of 234 *C. glabrata* isolates in this study.

| **Isolate** | **Year** | **Sex** | **Age** | **Sample** | **ST** | **CC** | **MIC (μg/mL)** | | | | | | | |
| --- | --- | --- | --- | --- | --- | --- | --- | --- | --- | --- | --- | --- | --- | --- |
|  |  |  |  |  |  |  | **AMB** | **FCT** | **MIF** | **CAS** | **FLU** | **VRC** | **POS** | **ITR** |
| RJ001 | 2019 | M | 27 | pus | 7 | 0 | 0.5 | ≤0.12 | ≤0.008 | 0.12 | 8 | 0.25 | 0.5 | 0.5 |
| RJ002 | 2019 | F | 86 | urine | 7 | 0 | 0.25 | ≤0.12 | ≤0.008 | 0.06 | 8 | 0.25 | 0.25 | 0.5 |
| RJ003 | 2019 | M | 89 | urine | 10 | 1 | 0.25 | ≤0.12 | ≤0.008 | 0.12 | 8 | 0.12 | 0.5 | 0.25 |
| RJ004 | 2019 | M | 92 | urine, catheter | 7 | 0 | 0.5 | ≤0.12 | ≤0.008 | 0.12 | 8 | 0.25 | 0.5 | 0.5 |
| RJ005 | 2019 | M | 50 | bile | 7 | 0 | 0.25 | ≤0.12 | ≤0.008 | 0.12 | 32 | 1 | 1 | 1 |
| RJ006 | 2019 | M | 64 | urine | 3 | 4 | 0.25 | ≤0.12 | ≤0.008 | 0.12 | 8 | 0.25 | 0.5 | 0.25 |
| RJ007 | 2019 | F | 86 | urine | 7 | 0 | 0.5 | ≤0.12 | ≤0.008 | 0.12 | 8 | 0.25 | 0.5 | 0.5 |
| RJ008 | 2021 | M | 63 | shunt fluid | 7 | 0 | 0.25 | ≤0.12 | ≤0.008 | 0.12 | 8 | 0.12 | 0.5 | 0.25 |
| RJ009 | 2019 | F | 43 | urine | 3 | 4 | 0.25 | ≤0.12 | ≤0.008 | 0.06 | 8 | 0.25 | 0.5 | 0.5 |
| RJ010 | 2019 | M | 60 | secretion | 7 | 0 | 0.5 | ≤0.12 | ≤0.008 | 0.12 | 8 | 0.12 | 0.5 | 0.5 |
| RJ011 | 2019 | M | 56 | blood | 10 | 1 | 0.25 | ≤0.12 | ≤0.008 | 0.12 | 8 | 0.25 | 0.5 | 0.5 |
| RJ012 | 2019 | F | 51 | urine | 7 | 0 | 0.5 | ≤0.12 | ≤0.008 | 0.12 | 8 | 0.25 | 0.5 | 0.5 |
| RJ013 | 2019 | M | 60 | shunt fluid | 7 | 0 | 1 | ≤0.12 | 0.03 | 0.25 | 4 | 0.12 | 0.5 | 0.25 |
| RJ014 | 2019 | F | 66 | urine | 10 | 1 | 0.25 | ≤0.12 | ≤0.008 | 0.06 | 8 | 0.12 | 0.5 | 0.25 |
| RJ015 | 2019 | F | 25 | urine | 7 | 0 | 0.25 | ≤0.12 | ≤0.008 | 0.12 | 8 | 0.25 | 0.5 | 0.5 |
| RJ016 | 2019 | F | 43 | urine | 7 | 0 | 0.25 | ≤0.12 | ≤0.008 | 0.12 | 8 | 0.12 | 0.25 | 0.5 |
| RJ017 | 2019 | F | 65 | urine | 7 | 0 | 2 | ≤0.12 | 0.03 | 0.25 | 16 | 0.5 | 1 | 1 |
| RJ018 | 2019 | F | 54 | urine | 7 | 0 | 0.25 | ≤0.12 | ≤0.008 | 0.25 | 4 | 0.12 | 0.25 | 0.25 |
| RJ019 | 2019 | M | 93 | urine | 7 | 0 | 0.5 | ≤0.12 | ≤0.008 | 0.12 | 8 | 0.25 | 0.5 | 0.5 |
| RJ020 | 2019 | F | 79 | urine | 10 | 1 | 0.25 | ≤0.12 | ≤0.008 | 0.12 | 8 | 0.12 | 0.5 | 0.5 |
| RJ021 | 2019 | F | 66 | urine | 7 | 0 | 0.25 | ≤0.12 | ≤0.008 | 0.06 | 8 | 0.25 | 0.5 | 0.5 |
| RJ022 | 2019 | F | 60 | urine | 10 | 1 | 1 | ≤0.12 | 0.03 | 0.25 | 4 | 0.12 | 0.5 | 0.25 |
| RJ023 | 2019 | M | 62 | urine | 7 | 0 | 0.25 | ≤0.12 | ≤0.008 | 0.25 | 64 | 2 | 16 | >16 |
| RJ024 | 2021 | M | 70 | bile | 7 | 0 | 0.25 | ≤0.12 | ≤0.008 | 0.12 | 16 | 0.25 | 0.5 | 0.5 |
| RJ027 | 2020 | M | 34 | catheter | 7 | 0 | 0.25 | ≤0.12 | ≤0.008 | 0.12 | 8 | 0.12 | 0.25 | 0.25 |
| RJ028 | 2019 | M | 71 | shunt fluid | 10 | 1 | 0.25 | ≤0.12 | ≤0.008 | 0.12 | 16 | 0.5 | 0.5 | 0.5 |
| RJ029 | 2019 | F | 67 | shunt fluid | 7 | 0 | 0.5 | ≤0.12 | ≤0.008 | 0.12 | 8 | 0.25 | 0.5 | 0.5 |
| RJ032 | 2021 | M | 64 | shunt fluid | 7 | 0 | 0.5 | ≤0.12 | ≤0.008 | 0.12 | 8 | 0.25 | 0.5 | 0.5 |
| RJ033 | 2019 | F | 55 | shunt fluid | 7 | 0 | 0.5 | ≤0.12 | ≤0.008 | 0.12 | 16 | 0.12 | 0.5 | 0.25 |
| RJ034 | 2021 | F | 73 | shunt fluid | 7 | 0 | 0.5 | ≤0.12 | ≤0.008 | 0.12 | 16 | 1 | 1 | 1 |
| RJ035 | 2020 | M | 58 | shunt fluid | 310 | 0 | 0.5 | ≤0.12 | ≤0.008 | 0.12 | 8 | 0.25 | 0.5 | 0.5 |
| RJ036 | 2021 | M | 91 | urine | 7 | 0 | 0.5 | ≤0.12 | ≤0.008 | 0.06 | 8 | 0.25 | 0.5 | 0.5 |
| RJ037 | 2019 | F | 91 | urine | 7 | 0 | 0.5 | ≤0.12 | ≤0.008 | 0.12 | 128 | 2 | >16 | >16 |
| RJ039 | 2020 | M | 75 | urine | 7 | 0 | 0.5 | ≤0.12 | ≤0.008 | 0.06 | 8 | 0.25 | 0.5 | 0.5 |
| RJ040 | 2020 | M | 75 | urine | 7 | 0 | 0.5 | ≤0.12 | ≤0.008 | 0.12 | 8 | 0.25 | 0.5 | 0.5 |
| RJ042 | 2020 | F | 76 | urine | 19 | 3 | 0.5 | ≤0.12 | ≤0.008 | 0.06 | 16 | 0.25 | 0.5 | 0.5 |
| RJ043 | 2020 | M | 69 | bile | 7 | 0 | 0.25 | ≤0.12 | ≤0.008 | 0.12 | 16 | 0.5 | 0.5 | 0.5 |
| RJ046 | 2022 | M | 77 | shunt fluid | 7 | 0 | 0.25 | ≤0.12 | ≤0.008 | 0.12 | 8 | 0.12 | 0.25 | 0.5 |
| RJ047 | 2022 | M | 52 | shunt fluid | 10 | 1 | 0.25 | ≤0.12 | ≤0.008 | 0.12 | 4 | 0.12 | 0.5 | 0.25 |
| RJ049 | 2022 | M | 24 | shunt fluid | 8 | 7 | 0.25 | ≤0.12 | ≤0.008 | 0.06 | 8 | 0.12 | 0.5 | 0.25 |
| RJ050 | 2022 | M | 77 | shunt fluid | 7 | 0 | 1 | ≤0.12 | ≤0.008 | 0.12 | 8 | 0.12 | 0.5 | 0.25 |
| RJ051 | 2022 | M | 53 | shunt fluid | 7 | 0 | 0.25 | ≤0.12 | ≤0.008 | 0.06 | 4 | 0.12 | 0.25 | 0.25 |
| RJ054 | 2022 | M | 73 | catheter | 7 | 0 | 0.25 | ≤0.12 | ≤0.008 | 0.12 | 8 | 0.25 | 0.25 | 0.25 |
| RJ055 | 2019 | F | 38 | vaginal secretion | 10 | 1 | 0.25 | ≤0.12 | ≤0.008 | 0.12 | 8 | 0.12 | 0.5 | 0.5 |
| RJ056 | 2019 | F | 78 | urine | 7 | 0 | 0.25 | ≤0.12 | ≤0.008 | 0.12 | 8 | 0.25 | 0.5 | 0.5 |
| RJ058 | 2019 | M | 94 | urine | 7 | 0 | 0.5 | ≤0.12 | ≤0.008 | 0.12 | 8 | 0.12 | 0.5 | 0.25 |
| RJ060 | 2019 | M | 93 | blood | 7 | 0 | 0.25 | ≤0.12 | ≤0.008 | 0.12 | 8 | 0.12 | 0.5 | 0.25 |
| RJ061 | 2019 | M | 82 | urine | 7 | 0 | 0.5 | ≤0.12 | ≤0.008 | 0.06 | 8 | 0.25 | 0.5 | 0.5 |
| RJ062 | 2020 | M | 76 | urine | 10 | 1 | 0.25 | ≤0.12 | ≤0.008 | 0.12 | 8 | 0.25 | 0.5 | 0.5 |
| RJ063 | 2020 | M | 82 | urine | 19 | 3 | 0.25 | ≤0.12 | ≤0.008 | 0.06 | 4 | 0.12 | 0.25 | 0.25 |
| RJ065 | 2020 | M | 86 | urine | 7 | 0 | 0.5 | ≤0.12 | ≤0.008 | 0.12 | 8 | 0.25 | 0.5 | 0.5 |
| RJ067 | 2020 | F | 29 | blood | 16 | 12 | 0.5 | ≤0.12 | ≤0.008 | 0.25 | 4 | 0.12 | 0.5 | 0.25 |
| RJ068 | 2020 | M | 64 | bile | 10 | 1 | 0.5 | ≤0.12 | ≤0.008 | 0.12 | 8 | 0.25 | 0.5 | 0.5 |
| RJ070 | 2020 | F | 85 | urine | 7 | 0 | 0.25 | ≤0.12 | 0.5 | 2 | 8 | 0.12 | 0.5 | 0.5 |
| RJ072 | 2020 | M | 73 | urine | 7 | 0 | 0.25 | 4 | ≤0.008 | 0.06 | 16 | 0.5 | 1 | 1 |
| RJ073 | 2020 | F | 30 | shunt fluid | 55 | 8 | 0.5 | ≤0.12 | ≤0.008 | 0.25 | 8 | 0.25 | 0.5 | 0.5 |
| RJ074 | 2020 | F | 66 | urine | 10 | 1 | 0.25 | ≤0.12 | ≤0.008 | 0.12 | 128 | 2 | 8 | >16 |
| RJ075 | 2020 | M | 73 | exudate | 7 | 0 | 0.25 | ≤0.12 | ≤0.008 | 0.12 | 16 | 0.5 | 1 | 0.5 |
| RJ077 | 2022 | M | 82 | shunt fluid | 7 | 0 | 0.25 | ≤0.12 | ≤0.008 | 0.25 | 8 | 0.12 | 0.5 | 0.25 |
| RJ078 | 2022 | M | 89 | anal swab | 7 | 0 | 0.25 | ≤0.12 | ≤0.008 | 0.12 | 64 | 2 | 1 | 2 |
| RJ079 | 2019 | M | 50 | catheter | 10 | 1 | 0.5 | ≤0.12 | ≤0.008 | 0.12 | 8 | 0.25 | 0.5 | 0.5 |
| RJ081 | 2019 | F | 51 | urine | 7 | 0 | 0.25 | ≤0.12 | ≤0.008 | 0.12 | 8 | 0.25 | 0.5 | 0.5 |
| RJ082 | 2019 | F | 51 | urine | 7 | 0 | 0.5 | ≤0.12 | ≤0.008 | 0.06 | 8 | 0.25 | 0.5 | 0.5 |
| RJ083 | 2019 | M | 60 | shunt fluid | 10 | 1 | 0.25 | ≤0.12 | ≤0.008 | 0.12 | 8 | 0.25 | 0.5 | 0.25 |
| RJ084 | 2019 | M | 61 | blood | 7 | 0 | 0.5 | ≤0.12 | ≤0.008 | 0.12 | 8 | 0.25 | 0.5 | 0.5 |
| RJ085 | 2019 | F | 59 | pus | 7 | 0 | 0.25 | ≤0.12 | ≤0.008 | 0.06 | 8 | 0.25 | 0.5 | 0.5 |
| RJ086 | 2019 | M | 29 | shunt fluid | 15 | 1 | 0.5 | ≤0.12 | ≤0.008 | 0.12 | 8 | 0.25 | 0.5 | 0.5 |
| RJ088 | 2019 | F | 50 | urine, catheter | 182 | 2 | 0.25 | ≤0.12 | ≤0.008 | 0.12 | 8 | 0.12 | 0.25 | 0.25 |
| RJ089 | 2019 | M | 46 | pus | 7 | 0 | 0.5 | ≤0.12 | ≤0.008 | 0.12 | 8 | 0.25 | 0.5 | 0.5 |
| RJ090 | 2020 | M | 35 | bile | 7 | 0 | 0.25 | ≤0.12 | ≤0.008 | 0.06 | 16 | 0.5 | 1 | 1 |
| RJ091 | 2020 | F | 76 | urine | 19 | 3 | 0.25 | ≤0.12 | ≤0.008 | 0.12 | 16 | 0.25 | 0.5 | 0.5 |
| RJ092 | 2020 | F | 55 | urine | 7 | 0 | 0.25 | ≤0.12 | ≤0.008 | 0.12 | 8 | 0.25 | 0.5 | 0.5 |
| RJ093 | 2020 | M | 86 | secretion | 7 | 0 | 0.25 | ≤0.12 | ≤0.008 | 0.12 | 8 | 0.25 | 0.5 | 0.5 |
| RJ094 | 2020 | M | 87 | urine | 7 | 0 | 0.25 | ≤0.12 | ≤0.008 | 0.12 | 8 | 0.25 | 0.5 | 0.5 |
| RJ095 | 2020 | M | 83 | urine | 307 | 0 | 0.5 | ≤0.12 | ≤0.008 | 0.12 | 8 | 0.25 | 0.5 | 0.5 |
| RJ096 | 2020 | F | 85 | urine, catheter | 7 | 0 | 0.25 | ≤0.12 | ≤0.008 | 0.12 | 8 | 0.25 | 0.5 | 0.5 |
| RJ097 | 2020 | F | 85 | urine | 7 | 0 | 0.25 | ≤0.12 | >8 | >8 | 8 | 0.12 | 0.5 | 0.25 |
| RJ098 | 2020 | M | 73 | urine | 7 | 0 | 0.25 | 2 | ≤0.008 | 0.12 | 16 | 0.25 | 0.5 | 0.5 |
| RJ099 | 2020 | M | 73 | urine | 7 | 0 | 0.5 | 4 | ≤0.008 | 0.12 | 16 | 0.5 | 1 | 1 |
| RJ100 | 2020 | M | 33 | shunt fluid | 15 | 1 | 0.5 | ≤0.12 | ≤0.008 | 0.12 | 8 | 0.25 | 0.5 | 0.5 |
| RJ101 | 2020 | F | 84 | urine | 7 | 0 | 0.25 | ≤0.12 | ≤0.008 | 0.06 | 64 | 1 | 1 | 2 |
| RJ102 | 2022 | F | 55 | shunt fluid | 7 | 0 | 0.5 | ≤0.12 | ≤0.008 | 0.12 | 8 | 0.25 | 0.5 | 0.5 |
| RJ103 | 2020 | M | 84 | urine | 7 | 0 | 0.25 | ≤0.12 | ≤0.008 | 0.12 | 8 | 0.12 | 0.5 | 0.25 |
| RJ105 | 2020 | F | 63 | urine | 7 | 0 | 0.25 | ≤0.12 | ≤0.008 | 0.12 | 8 | 0.12 | 0.25 | 0.25 |
| RJ106 | 2020 | F | 63 | urine | 7 | 0 | 0.25 | ≤0.12 | ≤0.008 | 0.06 | 4 | 0.12 | 0.25 | 0.25 |
| RJ107 | 2020 | F | 76 | urine | 10 | 1 | 0.5 | ≤0.12 | ≤0.008 | 0.12 | 4 | 0.12 | 0.25 | 0.25 |
| RJ108 | 2020 | F | 84 | urine | 7 | 0 | 0.25 | ≤0.12 | ≤0.008 | 0.12 | 32 | 1 | 2 | 2 |
| RJ109 | 2020 | M | 81 | shunt fluid | 7 | 0 | 0.25 | ≤0.12 | ≤0.008 | 0.12 | 8 | 0.25 | 0.5 | 0.5 |
| RJ111 | 2020 | F | 79 | urine, catheter | 7 | 0 | 0.5 | ≤0.12 | 0.12 | 0.5 | 64 | 2 | 4 | 2 |
| RJ112 | 2020 | M | 33 | pus | 15 | 1 | 0.5 | ≤0.12 | ≤0.008 | 0.12 | 8 | 0.25 | 0.5 | 0.5 |
| RJ113 | 2021 | M | 69 | urine | 55 | 8 | 0.25 | ≤0.12 | ≤0.008 | 0.12 | 8 | 0.25 | 0.5 | 0.5 |
| RJ114 | 2021 | M | 69 | urine, catheter | 55 | 8 | 1 | ≤0.12 | ≤0.008 | 0.12 | 8 | 0.12 | 0.5 | 0.5 |
| RJ115 | 2021 | F | 67 | urine | 3 | 4 | 0.5 | ≤0.12 | ≤0.008 | 0.12 | 8 | 0.25 | 0.5 | 0.5 |
| RJ118 | 2021 | F | 72 | urine | 182 | 2 | 0.25 | ≤0.12 | ≤0.008 | 0.12 | 8 | 0.25 | 0.5 | 0.5 |
| RJ120 | 2021 | M | 33 | shunt fluid | 15 | 1 | 0.5 | ≤0.12 | ≤0.008 | 0.12 | 8 | 0.25 | 0.5 | 0.5 |
| RJ121 | 2021 | M | 33 | catheter | 15 | 1 | 0.25 | ≤0.12 | ≤0.008 | 0.12 | 8 | 0.25 | 0.5 | 0.5 |
| RJ122 | 2021 | M | 33 | shunt fluid | 15 | 1 | 0.5 | ≤0.12 | ≤0.008 | 0.12 | 8 | 0.25 | 0.5 | 0.5 |
| RJ123 | 2021 | F | 86 | urine | 7 | 0 | 0.5 | ≤0.12 | ≤0.008 | 0.12 | 8 | 0.25 | 0.5 | 1 |
| RJ124 | 2021 | M | 36 | shunt fluid | 7 | 0 | 0.5 | ≤0.12 | ≤0.008 | 0.12 | 8 | 0.12 | 0.5 | 0.5 |
| RJ125 | 2021 | M | 79 | urine | 7 | 0 | 0.5 | ≤0.12 | ≤0.008 | 0.12 | 64 | 2 | 2 | 2 |
| RJ126 | 2021 | F | 87 | urine | 7 | 0 | 0.25 | ≤0.12 | ≤0.008 | 0.12 | 8 | 0.25 | 0.5 | 0.5 |
| RJ127 | 2022 | F | 82 | bile | 7 | 0 | 0.25 | ≤0.12 | ≤0.008 | 0.12 | 8 | 0.25 | 0.5 | 0.5 |
| RJ128 | 2022 | F | 82 | bile | 7 | 0 | 0.5 | ≤0.12 | ≤0.008 | 0.12 | 8 | 0.12 | 0.5 | 0.25 |
| RJ129 | 2022 | F | 82 | bile | 7 | 0 | 0.5 | ≤0.12 | ≤0.008 | 0.12 | 8 | 0.25 | 0.5 | 0.5 |
| RJ130 | 2022 | F | 82 | bile | 7 | 0 | 0.25 | ≤0.12 | ≤0.008 | 0.12 | 4 | 0.12 | 0.5 | 0.5 |
| RJ131 | 2020 | M | 79 | blood | 307 | 0 | 0.5 | ≤0.12 | ≤0.008 | 0.25 | 8 | 0.25 | 0.5 | 0.5 |
| RJ133 | 2021 | F | 65 | urine | 7 | 0 | 0.5 | ≤0.12 | ≤0.008 | 0.12 | 4 | 0.12 | 0.5 | 0.25 |
| RJ135 | 2021 | F | 65 | urine | 15 | 1 | 0.5 | ≤0.12 | ≤0.008 | 0.25 | 64 | 2 | >16 | >16 |
| RJ137 | 2021 | M | 75 | shunt fluid | 15 | 1 | 0.5 | ≤0.12 | ≤0.008 | 0.25 | 8 | 0.25 | 1 | 1 |
| RJ138 | 2021 | M | 36 | shunt fluid | 7 | 0 | 0.5 | ≤0.12 | ≤0.008 | 0.12 | 8 | 0.25 | 0.5 | 0.5 |
| RJ139 | 2021 | M | 79 | urine | 7 | 0 | 0.25 | ≤0.12 | ≤0.008 | 0.25 | 64 | 2 | >16 | >16 |
| RJ140 | 2021 | M | 79 | urine | 7 | 0 | 0.5 | ≤0.12 | ≤0.008 | 0.25 | 128 | 4 | >16 | >16 |
| RJ141 | 2021 | M | 34 | urine | 7 | 0 | 0.25 | 0.25 | ≤0.008 | 0.12 | 16 | 0.5 | 0.5 | 1 |
| RJ142 | 2021 | M | 72 | urine | 7 | 0 | 0.25 | ≤0.12 | ≤0.008 | 0.12 | 8 | 0.25 | 0.5 | 0.5 |
| RJ143 | 2021 | M | 75 | urine | 7 | 0 | 0.25 | ≤0.12 | ≤0.008 | 0.25 | 8 | 0.25 | 0.5 | 0.5 |
| RJ145 | 2021 | M | 36 | shunt fluid | 7 | 0 | 0.25 | ≤0.12 | ≤0.008 | 0.12 | 8 | 0.25 | 0.5 | 0.5 |
| RJ146 | 2021 | M | 34 | urine | 7 | 0 | 0.5 | 0.25 | ≤0.008 | 0.12 | 16 | 0.5 | 1 | 1 |
| RJ147 | 2021 | F | 74 | urine | 7 | 0 | 0.25 | ≤0.12 | ≤0.008 | 0.12 | 8 | 0.25 | 0.5 | 0.25 |
| RJ148 | 2021 | M | 34 | urine | 7 | 0 | 0.5 | 0.25 | ≤0.008 | 0.12 | 16 | 0.5 | 1 | 1 |
| RJ150 | 2021 | M | 34 | urine | 7 | 0 | 0.5 | 0.25 | ≤0.008 | 0.12 | 16 | 0.5 | 1 | 1 |
| RJ150 | 2021 | F | 80 | urine | 10 | 1 | 0.5 | 0.12 | ≤0.008 | 0.25 | 16 | 0.5 | 0.5 | 0.5 |
| RJ153 | 2021 | F | 68 | urine | 7 | 0 | 0.5 | ≤0.12 | ≤0.008 | 0.25 | 8 | 0.25 | 1 | 1 |
| RJ154 | 2021 | M | 55 | shunt fluid | 7 | 0 | 0.5 | ≤0.12 | ≤0.008 | 0.25 | 64 | 2 | >16 | >16 |
| RJ155 | 2021 | M | 87 | urine, catheter | 7 | 0 | 0.5 | ≤0.12 | ≤0.008 | 0.12 | 8 | 0.25 | 0.5 | 0.5 |
| RJ156 | 2021 | M | 79 | urine | 7 | 0 | 0.5 | ≤0.12 | ≤0.008 | 0.25 | 16 | 0.5 | 1 | 1 |
| RJ157 | 2021 | M | 87 | urine, catheter | 7 | 0 | 0.5 | ≤0.12 | ≤0.008 | 0.25 | 8 | 0.25 | 0.5 | 0.5 |
| RJ158 | 2021 | M | 72 | urine | 7 | 0 | 0.5 | ≤0.12 | ≤0.008 | 0.25 | 8 | 0.25 | 0.5 | 0.5 |
| RJ159 | 2021 | F | 47 | urine | 7 | 0 | 0.5 | ≤0.12 | ≤0.008 | 0.12 | 8 | 0.5 | 1 | 1 |
| RJ160 | 2021 | M | 40 | shunt fluid | 7 | 0 | 0.5 | ≤0.12 | ≤0.008 | 0.25 | 128 | 2 | >16 | >16 |
| RJ161 | 2021 | F | 74 | urine | 307 | 0 | 0.5 | ≤0.12 | ≤0.008 | 0.25 | 8 | 0.25 | 0.5 | 0.5 |
| RJ162 | 2021 | F | 82 | urine | 7 | 0 | 0.5 | ≤0.12 | 0.06 | 1 | 64 | 2 | 4 | >16 |
| RJ163 | 2021 | F | 86 | urine | 10 | 1 | 0.5 | ≤0.12 | 0.008 | 0.25 | 32 | 1 | 1 | 2 |
| RJ164 | 2021 | F | 61 | bile | 15 | 1 | 0.5 | ≤0.12 | 0.015 | 0.25 | 8 | 0.25 | 1 | 1 |
| RJ165 | 2021 | F | 36 | shunt fluid | 3 | 4 | 0.5 | 0.25 | ≤0.008 | 0.25 | 8 | 0.25 | 0.5 | 0.5 |
| RJ166 | 2021 | F | 71 | urine | 7 | 0 | 0.5 | ≤0.12 | ≤0.008 | 0.12 | 4 | 0.06 | 0.12 | 0.25 |
| RJ167 | 2021 | F | 59 | urine | 7 | 0 | 0.25 | ≤0.12 | ≤0.008 | 0.12 | 8 | 0.12 | 0.5 | 0.5 |
| RJ168 | 2021 | M | 84 | shunt fluid | 7 | 0 | 0.5 | ≤0.12 | ≤0.008 | 0.12 | 8 | 0.25 | 1 | 1 |
| RJ169 | 2021 | M | 63 | shunt fluid | 182 | 2 | 0.5 | ≤0.12 | ≤0.008 | 0.12 | 16 | 0.25 | 1 | 0.5 |
| RJ170 | 2021 | F | 62 | urine | 7 | 0 | 0.25 | ≤0.12 | ≤0.008 | 0.12 | 16 | 0.5 | 0.5 | 0.5 |
| RJ171 | 2021 | M | 84 | urine | 7 | 0 | 0.25 | ≤0.12 | ≤0.008 | 0.12 | 8 | 0.25 | 0.5 | 0.5 |
| RJ172 | 2021 | M | 70 | bile | 7 | 0 | 0.25 | ≤0.12 | ≤0.008 | 0.12 | 16 | 0.5 | 1 | 1 |
| RJ173 | 2022 | M | 64 | sputum | 7 | 0 | 0.5 | ≤0.12 | ≤0.008 | 0.12 | 8 | 0.25 | 0.5 | 0.5 |
| RJ174 | 2022 | M | 79 | sputum | 7 | 0 | 0.25 | ≤0.12 | ≤0.008 | 0.12 | 8 | 0.25 | 0.5 | 0.5 |
| RJ175 | 2022 | F | 89 | sputum | 7 | 0 | 0.25 | ≤0.12 | ≤0.008 | 0.25 | 64 | 1 | 1 | 2 |
| RJ176 | 2022 | F | 89 | sputum | 7 | 0 | 0.5 | ≤0.12 | ≤0.008 | 0.25 | 32 | 1 | 16 | >16 |
| RJ178 | 2022 | F | 89 | sputum | 7 | 0 | 0.5 | ≤0.12 | ≤0.008 | 0.25 | 128 | 4 | >16 | >16 |
| RJ179 | 2022 | F | 98 | sputum | 19 | 3 | 0.5 | ≤0.12 | ≤0.008 | 0.12 | 8 | 0.25 | 0.5 | 0.5 |
| RJ180 | 2022 | F | 89 | sputum | 7 | 0 | 0.5 | ≤0.12 | ≤0.008 | 0.12 | 64 | 1 | 1 | 2 |
| RJ181 | 2022 | F | 89 | sputum | 7 | 0 | 0.5 | ≤0.12 | ≤0.008 | 0.12 | 64 | 1 | 8 | 2 |
| RJ182 | 2022 | F | 89 | sputum | 7 | 0 | 0.5 | ≤0.12 | ≤0.008 | 0.25 | 64 | 2 | 2 | 2 |
| RJ183 | 2022 | F | 48 | sputum | 203 | Singleton | 0.5 | ≤0.12 | ≤0.008 | 0.12 | 8 | 0.25 | 0.5 | 0.25 |
| RJ185 | 2022 | F | 89 | sputum | 7 | 0 | 0.25 | ≤0.12 | ≤0.008 | 0.12 | 32 | 1 | 2 | 2 |
| RJ187 | 2022 | F | 89 | sputum | 7 | 0 | 0.25 | ≤0.12 | ≤0.008 | 0.12 | 128 | 2 | >16 | >16 |
| RJ188 | 2022 | F | 89 | sputum | 7 | 0 | 0.5 | ≤0.12 | ≤0.008 | 0.12 | 32 | 1 | 2 | 2 |
| RJ189 | 2022 | M | 88 | sputum | 10 | 1 | 0.25 | ≤0.12 | ≤0.008 | 0.12 | 8 | 0.5 | 1 | 1 |
| RJ190 | 2022 | F | 89 | sputum | 7 | 0 | 0.25 | ≤0.12 | ≤0.008 | 0.12 | 32 | 1 | 2 | 2 |
| RJ191 | 2022 | F | 89 | sputum | 7 | 0 | 0.5 | ≤0.12 | ≤0.008 | 0.12 | 64 | 2 | 8 | >16 |
| RJ192 | 2022 | M | 71 | sputum | 10 | 1 | 0.25 | ≤0.12 | ≤0.008 | 0.25 | 4 | 0.12 | 0.5 | 0.5 |
| RJ193 | 2022 | F | 36 | anal swab | 7 | 0 | 0.25 | ≤0.12 | ≤0.008 | 0.25 | 4 | 0.12 | 0.5 | 0.5 |
| RJ194 | 2022 | F | 89 | anal swab | 7 | 0 | 0.25 | ≤0.12 | ≤0.008 | 0.06 | 64 | 1 | 1 | 2 |
| RJ196 | 2022 | M | 62 | bile | 7 | 0 | 0.25 | ≤0.12 | ≤0.008 | 0.06 | 8 | 0.25 | 0.5 | 0.5 |
| RJ197 | 2022 | F | 36 | shunt fluid | 7 | 0 | 0.5 | ≤0.12 | ≤0.008 | 0.12 | 64 | 2 | 2 | 2 |
| RJ198 | 2022 | F | 72 | throat swab | 7 | 0 | 0.5 | ≤0.12 | ≤0.008 | 0.12 | 32 | 1 | 1 | 1 |
| RJ199 | 2022 | F | 36 | anal swab | 7 | 0 | 0.25 | ≤0.12 | ≤0.008 | 0.12 | 64 | 2 | 1 | 2 |
| RJ200 | 2022 | M | 69 | anal swab | 26 | 2 | 0.5 | ≤0.12 | ≤0.008 | 0.25 | 8 | 0.25 | 0.5 | 0.5 |
| RJ201 | 2022 | F | 84 | anal swab | 7 | 0 | 0.5 | ≤0.12 | ≤0.008 | 0.12 | 8 | 0.25 | 0.5 | 0.5 |
| RJ202 | 2022 | F | 84 | anal swab | 7 | 0 | 0.25 | ≤0.12 | ≤0.008 | 0.12 | 32 | 1 | 1 | 2 |
| RJ203 | 2022 | F | 89 | anal swab | 7 | 0 | 0.5 | ≤0.12 | ≤0.008 | 0.12 | 128 | 4 | >16 | >16 |
| RJ205 | 2022 | M | 67 | stool | 22 | 10 | 0.5 | ≤0.12 | ≤0.008 | 0.12 | 8 | 0.25 | 0.5 | 0.5 |
| RJ206 | 2022 | M | 74 | stool | 7 | 0 | 0.25 | ≤0.12 | ≤0.008 | 0.06 | 32 | 1 | 1 | 1 |
| RJ209 | 2022 | F | 74 | stool | 10 | 1 | 0.25 | ≤0.12 | ≤0.008 | 0.12 | 8 | 0.12 | 0.5 | 0.25 |
| RJ210 | 2022 | M | 90 | stool | 15 | 1 | 0.25 | ≤0.12 | ≤0.008 | 0.25 | 8 | 0.25 | 0.25 | 0.25 |
| RJ211 | 2023 | F | 63 | sputum | 7 | 0 | 0.5 | ≤0.12 | ≤0.008 | 0.12 | 8 | 0.25 | 0.25 | 0.5 |
| RJ212 | 2023 | M | 90 | anal swab | 7 | 0 | 0.5 | ≤0.12 | ≤0.008 | 0.12 | 32 | 1 | 1 | 2 |
| RJ213 | 2023 | F | 79 | anal swab | 7 | 0 | 0.5 | ≤0.12 | 0.03 | 0.25 | 8 | 0.5 | 1 | 1 |
| RJ214 | 2023 | F | 61 | stool | 10 | 1 | 0.5 | ≤0.12 | ≤0.008 | 0.25 | 16 | 0.5 | 0.5 | 0.5 |
| RJ215 | 2023 | F | 63 | sputum | 7 | 0 | 0.25 | ≤0.12 | ≤0.008 | 0.12 | 8 | 0.25 | 0.5 | 0.5 |
| RJ216 | 2023 | F | 85 | shunt fluid | 7 | 0 | 0.25 | ≤0.12 | ≤0.008 | 0.12 | 8 | 0.25 | 0.5 | 0.25 |
| RJ217 | 2023 | M | 72 | shunt fluid | 7 | 0 | 0.25 | ≤0.12 | ≤0.008 | 0.12 | 8 | 0.12 | 0.25 | 0.25 |
| RJ218 | 2023 | F | 89 | anal swab | 7 | 0 | 0.5 | ≤0.12 | ≤0.008 | 0.12 | 64 | 1 | 2 | 2 |
| RJ219 | 2023 | F | 48 | anal swab | 7 | 0 | 0.25 | ≤0.12 | ≤0.008 | 0.12 | 16 | 1 | >16 | >16 |
| RJ220 | 2023 | F | 89 | sputum | 7 | 0 | 0.5 | ≤0.12 | ≤0.008 | 0.12 | 128 | 4 | 16 | >16 |
| RJ221 | 2023 | M | 20 | throat swab | 3 | 4 | 0.25 | ≤0.12 | ≤0.008 | 0.12 | 64 | 2 | 1 | 1 |
| RJ222 | 2023 | M | 70 | anal swab | 7 | 0 | 0.5 | ≤0.12 | ≤0.008 | 0.12 | 8 | 0.12 | 0.5 | 0.5 |
| RJ223 | 2023 | F | 89 | anal swab | 7 | 0 | 0.5 | ≤0.12 | ≤0.008 | 0.12 | 64 | 2 | 2 | 2 |
| RJ224 | 2023 | F | 89 | sputum | 7 | 0 | 0.5 | ≤0.12 | ≤0.008 | 0.12 | 64 | 1 | 2 | 2 |
| RJ225 | 2023 | M | 93 | anal swab | 7 | 0 | 0.5 | ≤0.12 | ≤0.008 | 0.12 | 64 | 1 | 1 | 2 |
| RJ226 | 2023 | M | 37 | sputum | 7 | 0 | 0.5 | ≤0.12 | ≤0.008 | 0.06 | 8 | 0.25 | 0.5 | 0.5 |
| RJ227 | 2023 | M | 79 | sputum | 7 | 0 | 0.5 | ≤0.12 | ≤0.008 | 0.12 | 4 | 0.12 | 0.5 | 0.5 |
| RJ228 | 2023 | F | 30 | anal swab | 7 | 0 | 0.25 | ≤0.12 | ≤0.008 | 0.12 | 8 | 0.25 | 0.5 | 0.5 |
| RJ229 | 2023 | M | 66 | stool | 7 | 0 | 0.5 | ≤0.12 | ≤0.008 | 0.12 | 8 | 0.25 | 0.5 | 0.5 |
| RJ230 | 2023 | M | 73 | anal swab | 15 | 1 | 0.5 | ≤0.12 | ≤0.008 | 0.25 | 16 | 0.25 | 1 | 1 |
| RJ231 | 2023 | M | 66 | sputum | 3 | 4 | 0.5 | ≤0.12 | ≤0.008 | 0.12 | 8 | 0.12 | 0.5 | 0.5 |
| RJ232 | 2023 | M | 68 | bile | 15 | 1 | 0.5 | ≤0.12 | ≤0.008 | 0.12 | 16 | 0.5 | 1 | 1 |
| RJ233 | 2023 | M | 93 | anal swab | 7 | 0 | 0.5 | ≤0.12 | ≤0.008 | 0.12 | 64 | 1 | 2 | 2 |
| RJ234 | 2023 | M | 59 | anal swab | 311 | 0 | 0.5 | ≤0.12 | ≤0.008 | 0.12 | 4 | 0.06 | 0.25 | 0.25 |
| RJ235 | 2023 | F | 36 | anal swab | 7 | 0 | 0.5 | ≤0.12 | ≤0.008 | 0.25 | 32 | 1 | 2 | 2 |
| RJ236 | 2023 | M | 38 | stool | 7 | 0 | 0.5 | ≤0.12 | ≤0.008 | 0.12 | 8 | 0.25 | 0.5 | 0.5 |
| RJ237 | 2023 | M | 59 | anal swab | 311 | 0 | 0.5 | ≤0.12 | ≤0.008 | 0.12 | 4 | 0.12 | 0.25 | 0.25 |
| RJ238 | 2023 | M | 71 | sputum | 3 | 4 | 0.5 | ≤0.12 | ≤0.008 | 0.12 | 8 | 0.25 | 0.5 | 0.5 |
| RJ239 | 2023 | F | 59 | shunt fluid | 7 | 0 | 0.5 | ≤0.12 | ≤0.008 | 0.12 | 16 | 0.25 | 1 | 1 |
| RJ240 | 2023 | F | 53 | anal swab | 22 | 10 | 0.5 | ≤0.12 | ≤0.008 | 0.25 | 32 | 1 | 1 | 1 |
| RJ242 | 2023 | M | 93 | anal swab | 7 | 0 | 0.5 | ≤0.12 | ≤0.008 | 0.12 | 64 | 2 | 2 | 2 |
| RJ243 | 2023 | F | 67 | stool | 7 | 0 | 0.5 | ≤0.12 | ≤0.008 | 0.25 | 8 | 0.25 | 0.5 | 0.5 |
| RJ244 | 2023 | F | 85 | anal swab | 7 | 0 | 0.25 | ≤0.12 | ≤0.008 | 0.12 | 8 | 0.25 | 0.5 | 0.5 |
| RJ245 | 2023 | M | 38 | stool | 7 | 0 | 0.5 | ≤0.12 | ≤0.008 | 0.12 | 4 | 0.12 | 0.5 | 0.5 |
| RJ246 | 2023 | M | 37 | anal swab | 7 | 0 | 0.5 | ≤0.12 | ≤0.008 | 0.12 | 8 | 0.5 | 1 | 1 |
| RJ247 | 2023 | F | 36 | anal swab | 26 | 2 | 0.5 | ≤0.12 | ≤0.008 | 0.12 | 8 | 0.25 | 0.5 | 0.5 |
| RJ248 | 2023 | M | 68 | sputum | 7 | 0 | 0.25 | ≤0.12 | ≤0.008 | 0.12 | 8 | 0.25 | 0.5 | 0.5 |
| RJ249 | 2023 | F | 63 | wound | 7 | 0 | 0.5 | ≤0.12 | ≤0.008 | 0.25 | 8 | 0.25 | 0.5 | 0.5 |
| RJ250 | 2023 | M | 90 | anal swab | 7 | 0 | 0.25 | ≤0.12 | ≤0.008 | 0.06 | 4 | 0.12 | 0.25 | 0.25 |
| RJ251 | 2022 | F | 68 | sputum | 7 | 0 | 0.25 | ≤0.12 | ≤0.008 | 0.25 | 4 | 0.25 | 0.5 | 0.5 |
| RJ252 | 2022 | M | 79 | sputum | 7 | 0 | 0.5 | ≤0.12 | ≤0.008 | 0.06 | 8 | 0.25 | 0.5 | 0.5 |
| RJ253 | 2022 | F | 89 | sputum | 7 | 0 | 0.25 | ≤0.12 | ≤0.008 | 0.25 | 32 | 1 | 1 | 1 |
| RJ254 | 2022 | M | 54 | stool | 7 | 0 | 0.25 | ≤0.12 | ≤0.008 | 0.12 | 8 | 0.25 | 0.5 | 0.5 |
| RJ255 | 2022 | F | 24 | stool | 10 | 1 | 0.25 | ≤0.12 | ≤0.008 | 0.06 | 8 | 0.12 | 0.25 | 0.25 |
| RJ256 | 2022 | M | 72 | anal swab | 7 | 0 | 0.5 | ≤0.12 | ≤0.008 | 0.12 | 8 | 0.12 | 0.5 | 0.5 |
| RJ257 | 2022 | F | 57 | anal swab | 15 | 1 | 0.5 | ≤0.12 | ≤0.008 | 0.25 | 8 | 0.25 | 1 | 1 |
| RJ259 | 2023 | F | 61 | shunt fluid | 7 | 0 | 0.25 | ≤0.12 | ≤0.008 | 0.12 | 8 | 0.12 | 0.5 | 0.5 |
| RJ260 | 2023 | F | 36 | anal swab | 7 | 0 | 0.5 | ≤0.12 | ≤0.008 | 0.12 | 32 | 1 | 2 | 2 |
| RJ261 | 2022 | M | 69 | stool | 7 | 0 | 0.25 | ≤0.12 | ≤0.008 | 0.25 | 8 | 0.25 | 0.5 | 0.5 |
| RJ262 | 2023 | F | 36 | anal swab | 7 | 0 | 0.5 | ≤0.12 | ≤0.008 | 0.06 | 32 | 1 | 1 | 2 |
| RJ263 | 2023 | M | 71 | shunt fluid | 10 | 1 | 0.25 | ≤0.12 | ≤0.008 | 0.12 | 8 | 0.12 | 0.5 | 0.25 |
| RJ264 | 2023 | M | 66 | sputum | 3 | 4 | 0.5 | ≤0.12 | ≤0.008 | 0.12 | 8 | 0.12 | 0.5 | 0.5 |
| RJ266 | 2023 | M | 58 | sputum | 3 | 4 | 0.5 | ≤0.12 | ≤0.008 | 0.12 | 8 | 0.12 | 0.5 | 0.5 |
| RJ267 | 2023 | M | 93 | sputum | 7 | 0 | 0.5 | ≤0.12 | ≤0.008 | 0.12 | 64 | 2 | 2 | 2 |
| RJ268 | 2023 | M | 49 | bile | 7 | 0 | 0.25 | ≤0.12 | ≤0.008 | 0.25 | 8 | 0.12 | 0.5 | 0.25 |
| RJ269 | 2023 | M | 61 | sputum | 7 | 0 | 0.25 | ≤0.12 | ≤0.008 | 0.12 | 8 | 0.25 | 0.5 | 0.5 |
| RJ270 | 2023 | M | 68 | catheter | 15 | 1 | 0.5 | ≤0.12 | 1 | 8 | 8 | 0.12 | 0.5 | 0.5 |
| RJ273 | 2023 | F | 89 | sputum | 7 | 0 | 0.5 | ≤0.12 | ≤0.008 | 0.12 | 128 | 4 | 16 | >16 |
| RJ274 | 2023 | M | 60 | sputum | 195 | 5 | 0.5 | ≤0.12 | ≤0.008 | 0.12 | 4 | 0.06 | 0.25 | 0.25 |
| RJ275 | 2023 | F | 48 | anal swab | 7 | 0 | 0.5 | ≤0.12 | ≤0.008 | 0.12 | 16 | 1 | 16 | >16 |
| RJ276 | 2023 | M | 60 | stool | 195 | 5 | 0.5 | ≤0.12 | ≤0.008 | 0.12 | 4 | 0.06 | 0.25 | 0.25 |
| RJ277 | 2023 | F | 36 | anal swab | 7 | 0 | 0.5 | ≤0.12 | ≤0.008 | 0.12 | 32 | 1 | 2 | 2 |

**Abbreviations**: F, female; M, male; ST, sequence type; CC, clonal complex; MIC, minimum inhibitory concentration; AMB, amphotericin B; FCT, 5-flucytosine; MIF, micafungin; CAS, caspofungin; FLU, fluconazole; VRC, voriconazole; POS, **posaconazole**; ITR, itraconazole.
